# Endometrial organoids derived from Mayer–Rokitansky–Küster–Hauser syndrome patients provide insights into disease-causing pathways

**DOI:** 10.1242/dmm.049379

**Published:** 2022-05-10

**Authors:** Sara Y. Brucker, Thomas Hentrich, Julia M. Schulze-Hentrich, Martin Pietzsch, Noel Wajngarten, Anjali Ralhan Singh, Katharina Rall, André Koch

**Affiliations:** 1 Department of Women's Health, University of Tübingen, 72076 Tübingen, Germany; 2 Rare Disease Center Tübingen, University of Tübingen, 72076 Tübingen, Germany; 3 Institute of Medical Genetics and Applied Genomics, University of Tübingen, 72076 Tübingen, Germany; 4 Institute for Bioinformatics and Medical Informatics (IBMI), University of Tübingen, 72076 Tübingen, Germany; 5 Research Institute for Women's Health, University of Tübingen, 72076 Tübingen, Germany

**Keywords:** Developmental biology, Müllerian ducts, MRKH syndrome, RNA sequencing, Patient-derived organoids

## Abstract

The uterus is responsible for the nourishment and mechanical protection of the developing embryo and fetus and is an essential part in mammalian reproduction. Mayer–Rokitansky–Küster–Hauser (MRKH) syndrome is characterized by agenesis of the uterus and upper part of the vagina in females with normal ovarian function. Although heavily studied, the cause of the disease is still enigmatic. Current research in the field of MRKH mainly focuses on DNA-sequencing efforts and, so far, has been unable to decipher the nature and heterogeneity of the disease, thereby holding back scientific and clinical progress. Here, we developed long-term expandable organoid cultures from endometrium found in uterine rudiment horns of MRKH patients. Phenotypically, they share great similarity with healthy control organoids and are surprisingly fully hormone responsive. Transcriptome analyses, however, identified an array of dysregulated genes that point to potentially disease-causing pathways altered during the development of the female reproductive tract. We consider the endometrial organoid cultures to be a powerful research tool that promise to enable an array of studies into the pathogenic origins of MRKH syndrome and possible treatment opportunities to improve patient quality of life.

## Abstract (precis)

**Summary:** Müllerian agenesis is highly understudied due to the lack of experimental model systems. We close a significant gap by providing the first patient-derived organoid model of diseased tissue.

## INTRODUCTION

Mayer–Rokitansky–Küster–Hauser (MRKH) syndrome (OMIM: 277000) is a rare malformation, characterized by the partial or complete absence of the uterus and the upper two-thirds of the vagina, due to a still unknown defect in embryonic development (Oppelt et al., 2006). It affects 1 in 4500 women, making it the second most common reason for primary amenorrhea (Aittomäki et al., 2001; Herlin et al., 2016; Timmreck and Reindollar, 2003). Typically, MRKH patients have a normal female karyotype (46, XX) and regular development of secondary sexual characteristics, as their ovaries are functional. Although MRKH syndrome can occur as an isolated genital malformation (MRKH Type I), it is often associated with additional renal and/or skeletal, and to a lesser extent, with auditory, cardiac and other defects (MRKH Type II) (Oppelt et al., 2012; Rall et al., 2015b). In both cases, patients with MRKH frequently have one or two uterine remnants, consisting of myometrium and, although less often, also endometrium (Rall et al., 2013).

The etiology of the syndrome remains largely enigmatic, yet the spectrum of malformations encountered in MRKH patients suggests that the disease originates from a developmental defect during embryogenesis. Moreover, cases of familial clustering have implied a genetic component in the etiology (Herlin et al., 2014; Williams et al., 2017; Nik-Zainal et al., 2011). Mutations in *WNT4*, which is essential for the complete formation of the Müllerian ducts (MDs) (Vainio et al., 1999), have been reported earlier as a possible cause for MRKH in a small number of patients (Biason-Lauber et al., 2004; Philibert et al., 2008, 2011). Since *WNT4* is also necessary to prevent formation of Leydig cells in women, patients with mutated *WNT4* also present with clinical hyperandrogenism, rendering it a slightly different clinical entity (Biason-Lauber et al., 2004, 2007; Philibert et al., 2008). Furthermore, in some patients, mutations and possibly harmful variants have been found in developmental genes like *WNT9B* (Wang et al., 2014; Waschk et al., 2016), *LHX1* (Ledig et al., 2011, 2012; Sandbacka et al., 2013) or *TBX6* (Sandbacka et al., 2013; Tewes et al., 2015, 2019). Of high interest are specifically *LHX1*, *WNT4* and *WNT9B* due to their previously reported role in the formation of the MDs from the coelomic epithelium in gestational week 6 (Mullen and Behringer, 2014). After the two MDs are formed, they start growing caudally along the Wolffian ducts (WDs). By week 8, the terminal ends of the MDs begin to fuse, forming the uterovaginal duct. In males, the MDs start to regress after week 10 under the influence of anti-Müllerian hormone (AMH) and WNT7A. In females, however, they differentiate into the fallopian tubes, uterus, cervix and vagina, specifically regulated by the coordinated action of transcription factors and signaling molecules such as homeodomain transcription factors (e.g. Hox genes) and members of the WNT family (Robboy et al., 2017; Roly et al., 2018). This concerted interplay between transcription factors, hormones and growth signals during embryogenesis leads to a variety of highly timed and spatial gene expression changes (Roly et al., 2020). The widespread lack of a clear genetic link in MRKH syndrome suggests that the answer might be found at the transcriptional level. Therefore, the focus in recent years has shifted towards molecular characterizations of primary diseased tissue, identifying a plethora of new candidate genes and regulatory networks that might drive or contribute to the pathology (Hentrich et al., 2020; Rall et al., 2015a). In addition, hormonal stimulation of primary endometrial stroma cultures derived from MRKH rudimentary tissue showed a reduced transcriptional response compared to healthy controls (Brucker et al., 2017), indicating that dysfunctional hormone receptors play a role in the pathophysiology of MRKH (Rall et al., 2013). However, as other estrogen- and progesterone-dependent tissues like the breast develop normally in MRKH patients, any possible defect would have to be limited to the genital tract. This stresses the importance of studying the rudimentary tissue directly. Whereas stromal cultures have already been investigated (Brucker et al., 2017), the absence of a functional model for the glandular epithelium of MRKH endometrium to analyze the characteristics and capabilities of affected cell types vastly limited the pathophysiological understanding of this disease. Recent years have seen the establishment of patient-derived organoid models from healthy and diseased endometrium (Turco et al., 2017; Boretto et al., 2017, 2019). Organoids are self-renewing, three-dimensional (3D) models that mimic key properties and characteristics of the original *in vivo* tissue, which greatly facilitates research into complex interactions of the involved cell types.

Here, we show for the first time the successful establishment of organoid models from endometrium found in the rudimentary tissue of MRKH patients. The established organoids showed high similarity to healthy endometrial organoids, were hormone responsive and could be cultured long term. Yet, gene expression analyses by RNA sequencing showed distinctive differences between diseased and healthy organoids, emphasizing their potential as a powerful tool to investigate the etiology of MRKH syndrome. We identified several important developmental transcription factors to be differentially expressed between healthy and MRKH organoids. This highlights the importance and value of these models for studying and understanding the pathogenesis. The establishment and long-term growth of epithelial tissue from MRKH rudiments provides a powerful addition to the toolbox for studying the disease in a controlled and standardized environment, and these endometrial organoids promise to pave new avenues towards a better understanding and possible treatments of the disease.

## RESULTS

### Long-term 3D organoid cultures can be established from uterine rudiment endometrium

**Fig. 1.**
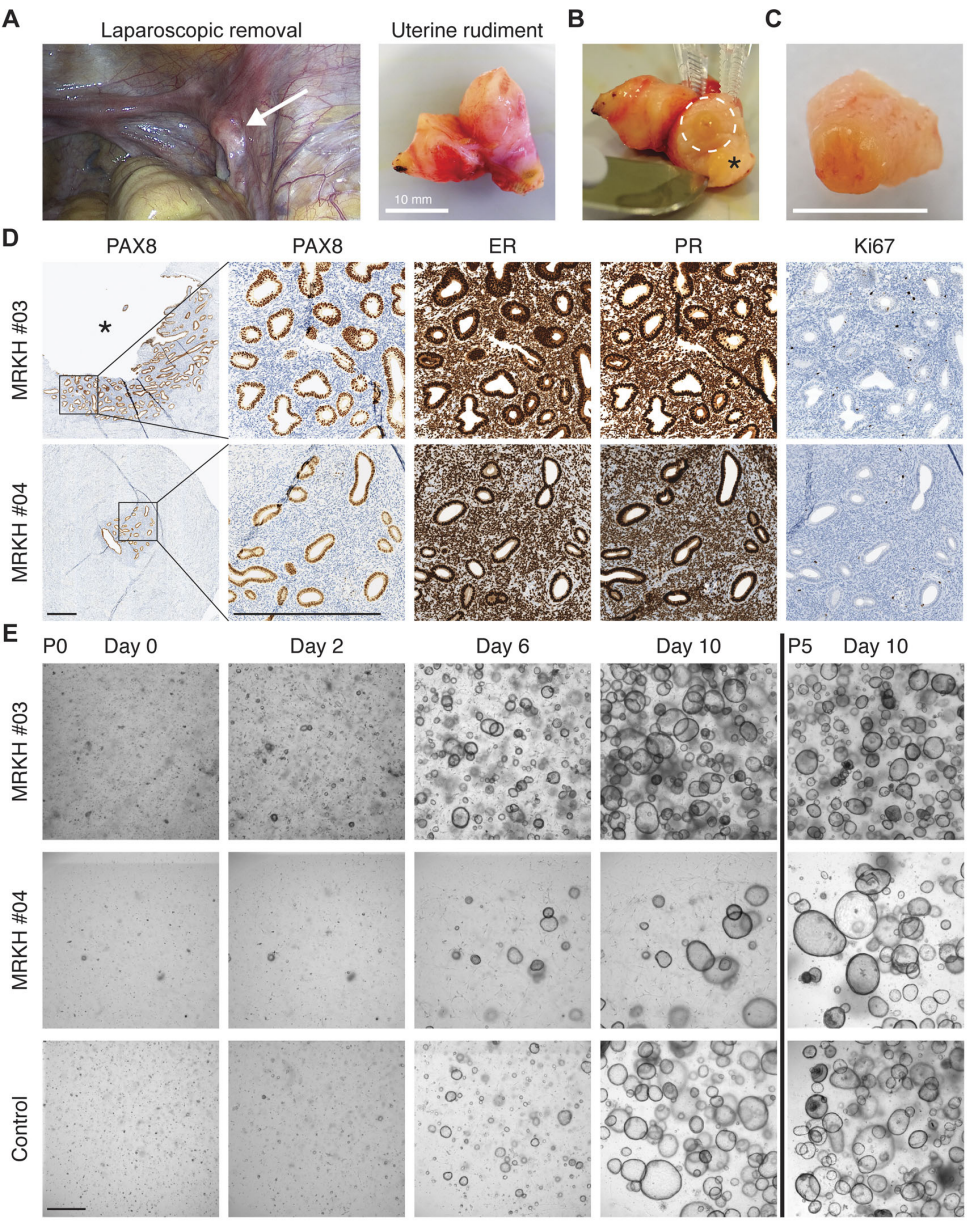
**Long-term 3D organoid cultures can be established from uterine rudiment endometrium.** (A) Left: laparoscopic image of an 18-year-old patient with MRKH syndrome (MRKH #03). White arrow indicates the uterine rudiment horn. Right: laparoscopically excised uterine rudiment horn before segmentation. Scale bar: 10 mm. (B) Sectioned uterine rudiment horn of MRKH #03. White dashed line circle depicts macroscopic endometrium used for organoid establishment (see C). Asterisk indicates the part that underwent pathological characterization (see corresponding pictures in D). (C) Excised section of macroscopical endometrium used for digestion and organoid establishment. Scale bar: 10 mm. (D) Immunohistochemical characterization of uterine rudiment from MRKH patient #03 (top row) and MRKH patient #04 (bottom row). Analyzed were PAX8, estrogen receptor alpha (ER), progesterone receptor (PR) and proliferation marker Ki67. The asterisk in MRKH #03 indicates the region marked in B. Both models show endometrial gland structures (PAX8-positive) with widespread and intense ER and PR expression in glandular and stromal compartments. There is almost no proliferation capacity visible in both MRKH tissues. Scale bars: 500 µm. (E) Bright-field images of cell suspensions from endometrial MRKH tissue digestions (top and middle rows) as well as from a healthy control (bottom row) after seeding (P0). Organoid growth for the same spot on the culture plate was monitored over the course of 10 days (Day 0–10). The right panel shows the same cultures at day 10 of the fifth passage (P5). Scale bar: 500 µm.

Human endometrial organoid cultures have recently been established by several research groups (Turco et al., 2017; Boretto et al., 2019, 2017). Typically, glandular structures obtained after processing endometrial biopsies are embedded in an extracellular matrix (ECM) component such as basement membrane extract (BME) or Matrigel and cultured in the presence of a defined cocktail of growth factors (Table S2). Since endometrium can be found in MRKH uterine rudiments (Rall et al., 2013), we screened a cohort of MRKH patients that underwent a laparoscopically assisted creation of a neovagina for the presence of endometrium in uterine rudiments. A total of 48 patients [35 MRKH Type I (73%), 13 MRKH Type II (27%)] were screened, of which 37 [32/35 Type I (91%), 5/13 Type II (38%)] had uterine rudiments present (Fig. 1A). We were able to macroscopically detect endometrium (Fig. 1B,C) in 12 patients [12/32 Type I (33%), 0/5 Type II (0%)]. Endometrium of four rudiments was also subjected to histological processing (Fig. 1B, asterisk) and immunohistochemistry for the transcription factor PAX8 (highly expressed in endometrial epithelial cells), estrogen receptor alpha (herein referred to as ER; encoded by *ESR1*), progesterone receptor (PR; encoded by *PGR*) and the proliferation marker Ki67 (also known as MKI67) (Fig. 1D) to verify the endometrium entity of the tissue. PAX8 staining confirmed that the endometrial epithelium of MRKH patients had all the morphological features of a normal endometrium, showing tubular and frequently branching glands with a single layer of columnar epithelium (Fig. 1D), as it has been shown recently in an extensive histology study of MRKH rudiments (Rall et al., 2013). Whereas ER and PR were ubiquitously expressed in the epithelial as well as stromal cells of the endometrium, the proliferative capacity, measured by Ki67 expression, was almost absent in the epithelium and stroma of MRKH patients (Fig. 1D), as reported previously (Rall et al., 2013). Although the proliferative capacity of the initial cell population was low, we successfully established 14 organoid models (ten patients had endometrium present unilaterally and two patients bilaterally, which accounts for the two additional organoid models) (Table S1). The success rate in establishing organoid models from patients with a macroscopically detected endometrium was 100% (14/14). The fact that the endometrium size of MRKH patients ranged from a few millimeters in diameter (e.g. MRKH #03, Fig. 1D) to even under a millimeter (e.g. MRKH #04, Fig. 1D) limited the amount of starting material for culture setup, but did not hinder the successful establishment of an organoid model (compare MRKH #03 and #04, Fig. 1E). Within 3–6 days, cystic organoid structures were visible, and all 14 established MRKH organoid models as well as four control models (Fig. 1E; Fig. S1A,B) were successfully cultured long term for more than 15 passages (>6 months of culture) without showing signs of a decrease in proliferation (Fig. S1C). Notably, the organoid models could easily be cryopreserved and stored as a live biobank (see Materials and Methods).

### MRKH organoids show high phenotypic similarity to organoids from healthy controls

**Fig. 2.**
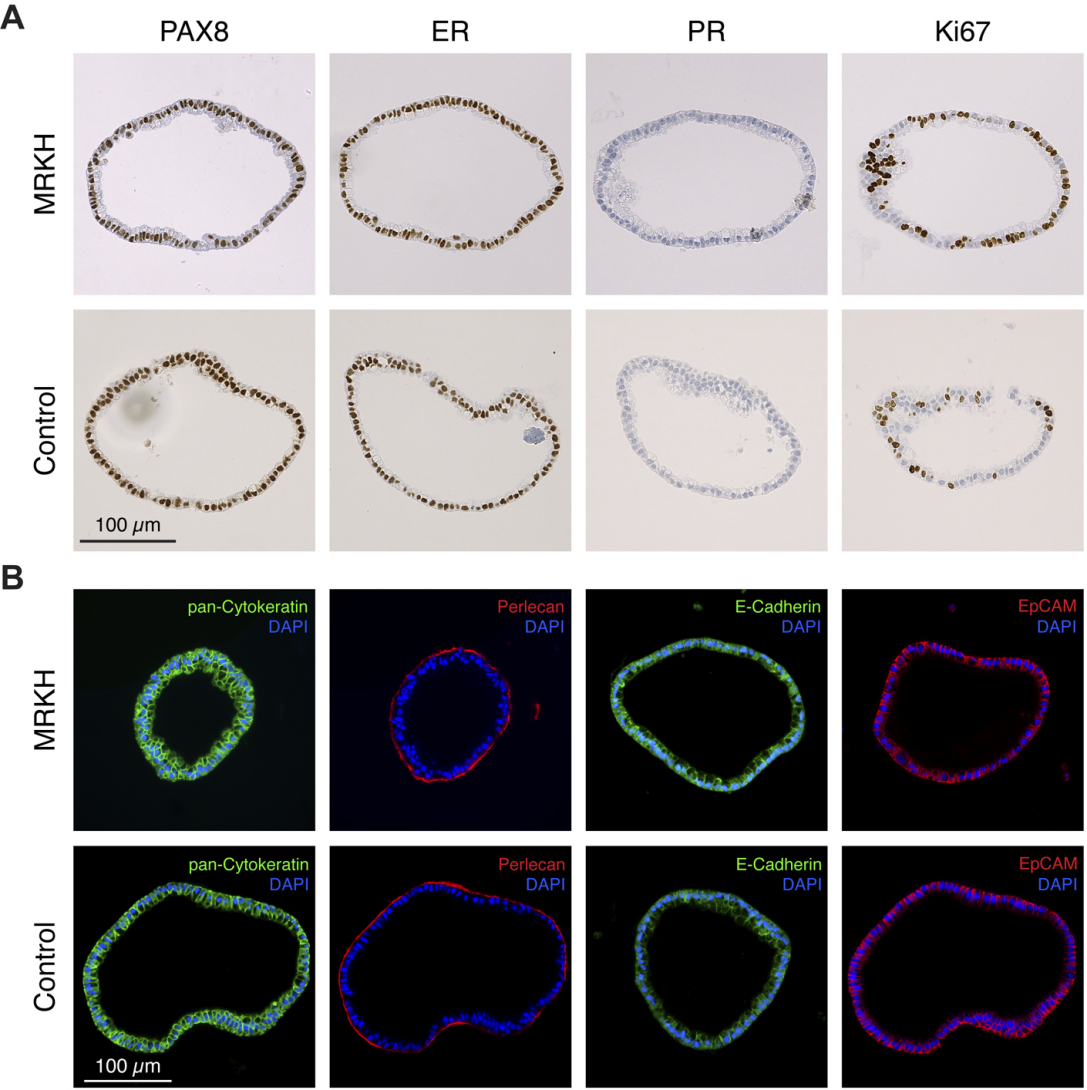
**MRKH endometrial organoids show high phenotypic similarity to endometrial organoids from healthy controls.** (A) Representative immunohistochemistry images of sections from FFPE MRKH (top row) and control (bottom row) endometrial organoids. Stained were the transcription factor PAX8, estrogen receptor alpha (ER), progesterone receptor (PR) and proliferation marker Ki67. Scale bar: 100 µm. (B) Representative immunofluorescence images of sections from FFPE MRKH (top row) and control (bottom row) endometrial organoids. The epithelial origin of organoids is shown by pan-cytokeratin (green), E-Cadherin (green) and EpCAM (red); epithelial polarity is shown by Perlecan (red). In all instances DAPI (blue) was used as counterstain for nuclei. Scale bar: 100 µm. Images are representative of at least 100 organoids (*n*=1).

The endometrial organoids of MRKH patients and healthy controls were expanded and passaged at ratios of 1:2 or 1:3 every 10–14 days and characterized by immunohistochemistry and immunofluorescence (Fig. 2A). Similar to the primary tissue, the endometrial organoids showed high and ubiquitous expression of PAX8 and ER, whereas the absence of steroid hormones (e.g. estradiol) in the culture medium explains the absence of PR expression in all models. The addition of estradiol to the culture medium led to the restoration of PR expression in both control and MRKH organoids (Fig. S1D). In sharp contrast to the observations in MRKH endometrial tissue, the organoids of MRKH patients expressed high levels of the proliferation marker Ki67, comparable to healthy controls. Markers of glandular epithelium [cytokeratin, epithelial cell adhesion molecule (EpCAM) and E-Cadherin] were ubiquitously expressed in all organoid models, and Perlecan staining at the basolateral membrane throughout the entirety of the endometrial organoids showed that epithelial polarity remained intact (Fig. 2B). Interestingly, phenotypic and morphological characterizations of healthy and diseased organoid models revealed no obvious differences.

### Organoids derived from MRKH patients differ transcriptionally from healthy controls

**Fig. 3.**
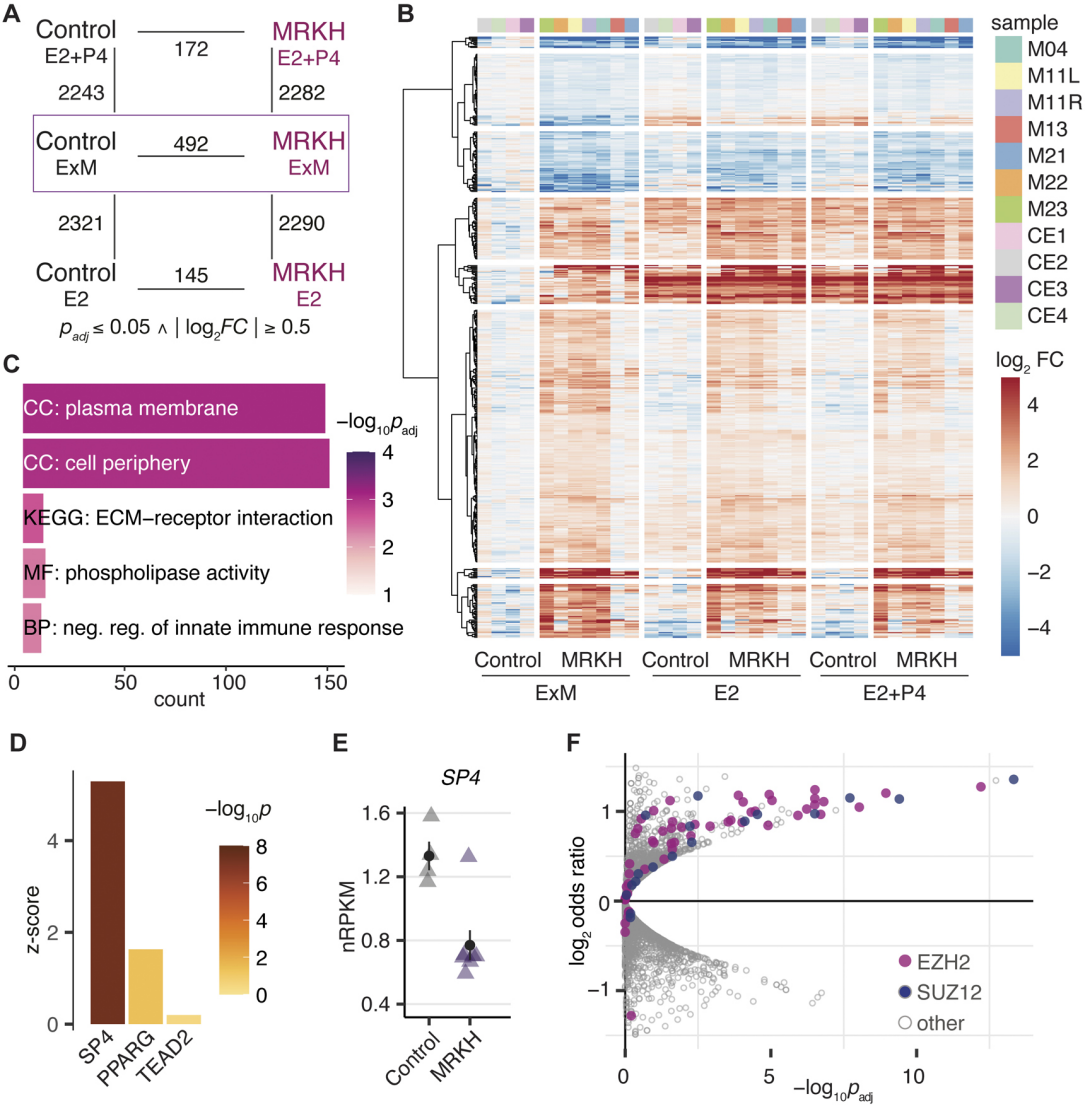
**Organoids derived from MRKH patients and healthy controls differ transcriptionally.** (A) Diagram of experimental groups and number of differentially expressed genes (DEGs) between pairwise comparisons according to the indicated fold change (|log_2_FC|) and significance (*P*_adj_) cut-offs. Control, organoids derived from unaffected women; MRKH, organoids derived from MRKH patients; ExM, organoids grown in expansion medium; E2, organoids treated with beta-estradiol; E2+P4, organoids treated with beta-estradiol and progesterone. (B) Expression profiles (log_2_ expression change relative to the control ExM group) of 492 DEGs across all samples. Rows are hierarchically clustered by Euclidian distance and the ‘ward.D2’ clustering method. Patient origin is color-coded on top. (C) Enrichment analysis of overrepresented Gene Ontology and KEGG terms for 492 DEGs (identified in MRKH/Control in ExM). Top five most significant terms with number of associated genes shown. CC, cellular compartment; MF, molecular function; BP, biological process. (D) Transcription factor-binding site analysis of 492 DEGs. Depicted are the top three scoring position weight matrices of transcription factors that are also differentially expressed. Higher z-scores reflect higher enrichment of the binding motif among DEGs. (E) Expression levels for *SP4* plotted as individual data points, the mean±s.e.m. is shown (*n*=4 for control, *n*=7 for MRKH). (F) Enrichment analysis of transcriptional regulators for 492 DEGs identified in MRKH organoids based on ChIP-seq and DNase I data according to TFEA.ChIP. *EZH2* and *SUZ12* are predicted to be significantly overrepresented among DEGs relative to the genome. Analysis is based on default parameters for binding sites <1 kB upstream including enhancer elements. Each dot represents a ChIP-seq accession, *EZH2*- and *SUZ12*-related accessions are shown in pink and purple, respectively.

After investigating organoid morphology, we next interrogated their transcriptome. Using RNA sequencing, we profiled seven organoid models from MRKH patients and four from healthy controls that were grown in expansion medium (ExM), treated with beta-estradiol (E2) or the combination of beta-estradiol and progesterone (E2+P4). Based on these six experimental groups, differential gene expression was determined according to disease status and treatment with cut-offs of Benjamini–Hochberg-adjusted *P*-value (*P*_adj_)≤0.05 and |log_2_ fold-change (FC)|≥0.5 (Fig. 3A; Table S6).

In the principal component analysis (PCA) of expression profiles, the samples partitioned clearly along principal components 1 (PC1) and 2 (PC2). The grouping along PC1 into ExM-treated samples to the right and hormone-treated samples to the left indicated the strongest influence on expression differences through treatment (Fig. S2). Accordingly, the expression effects of E2 and E2+P4 treatment led to about five times more differentially expressed genes (DEGs) compared to disease status comparisons, which yielded 492 DEGs for ExM treatment (Fig. 3A). The PCA further suggested great homogeneity in organoid responses with respect to treatment as reflected by the sample partitioning along PC2 according to patient origin (Fig. S2). Moreover, computational estimation of the underlying cell-type composition through single-cell reference data from uterus (Wang et al., 2020) and endometrial epithelial organoids (Fitzgerald et al., 2019) showed very consistent signatures across all samples with highest expression for epithelial cell types, agreeing with the model and in line with immunostainings for epithelial markers (Fig. 2B; Figs S3 and S4).

### Transcriptional changes are partially shared between MRKH organoids and primary patient tissue

Focusing on differential expression under ExM first, we identified 492 DEGs with 365 upregulated and 127 downregulated genes (Fig. 3B). Among them were 12 homeobox proteins (upregulated in MRKH: *LHX1*, *HOXD8*, *ONECUT3*, *LBX2*, *HOXB4*, *SATB1* and *HOXB6*; downregulated in MRKH: *EMX2*, *ZHX3*, *IRX3*, *NKX6-2* and *IRX5*). Of those, *LHX1*, *EMX2* and the Hox genes have previously been associated with MRKH syndrome either by sequencing of patient blood or functional *in vivo* studies with animal models (Ledig et al., 2012; Masse et al., 2009; Miyamoto et al., 1997). Moreover, supporting the role of the 492 DEGs in MD development, a large proportion of these genes are specifically activated during duct morphogenesis in chicken (Fig. S5) (Roly et al., 2020).

Applying enrichment analyses to identify affected pathways and cellular processes as well as transcriptional regulators potentially driving the differential expression, we identified ‘plasma membrane’ and ‘cell periphery’ as the most significant Gene Ontology (GO) terms and ‘ECM-receptor interaction’ with respect to the Kyoto Encyclopedia of Genes and Genomes (KEGG) database (Fig. 3C). A binding site analysis suggested highly significant overrepresentation of the *SP4* motif among DEG promotors (Fig. 3D). Interestingly, expression of *SP4* was decreased in MRKH organoids (Fig. 3E), agreeing with previous observations of a smaller uterus in *Sp4*-knockout mice (Göllner et al., 2001). Complementary analyses that utilized chromatin immunoprecipitation-sequencing (ChIP-seq) data and thereby also accounted for indirect binding events, as well as transcription factors with less clear motifs (Puente-Santamaria et al., 2019), suggested the DEG set to be enriched for *EZH2* and *SUZ12* targets (Fig. 3F). Since *EZH2* was also identified in our previous study of MRKH endometrial tissue (Hentrich et al., 2020), we saw accumulating evidence that further suggested epigenetic investigations into the origins of the syndrome.

**Fig. 4.**
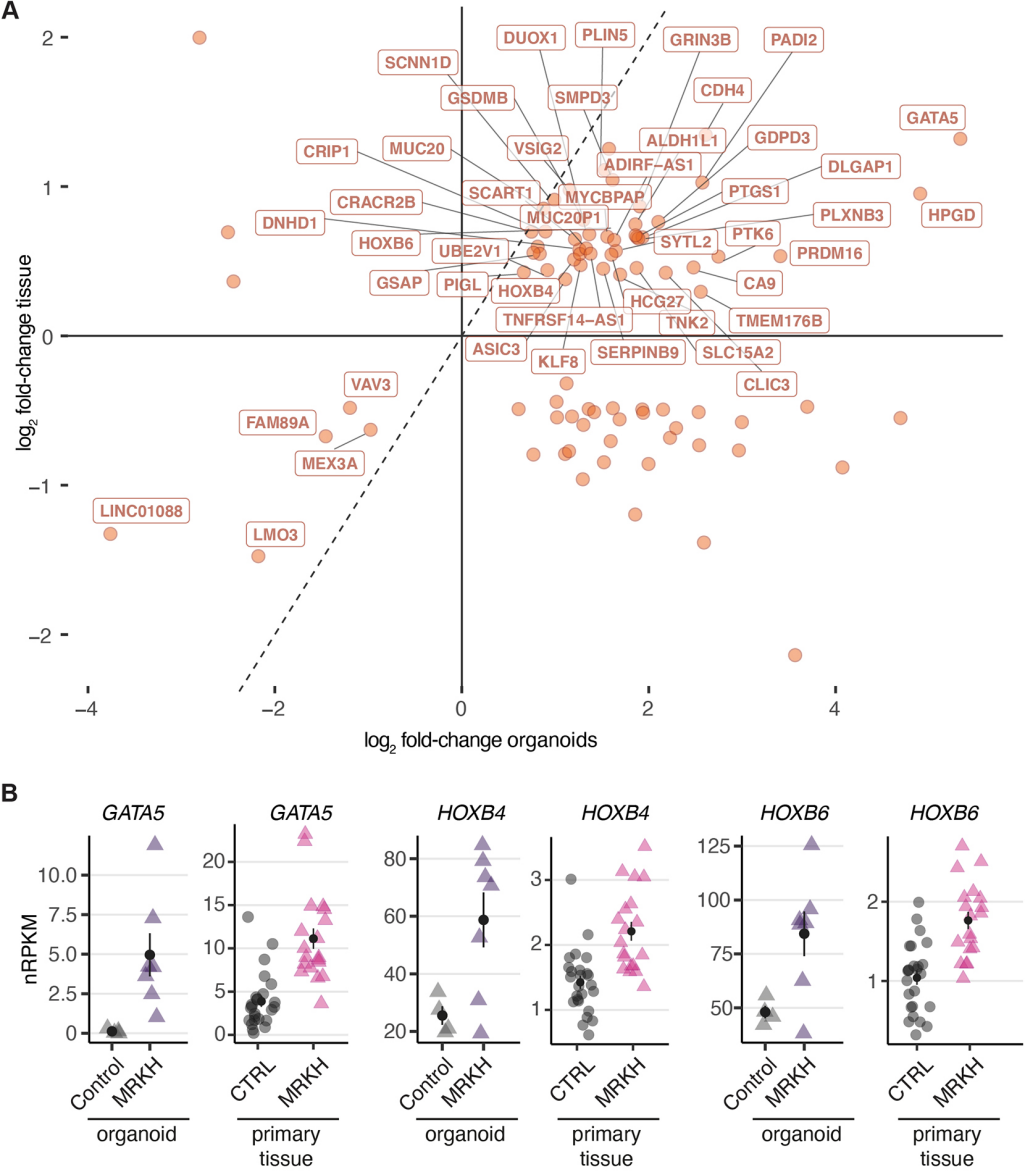
**MRKH organoids share expression changes with endometrial tissue samples.** (A) Scatter plot of 86 common DEGs between MRKH organoids and endometrial patient tissue. DEGs with directional similarity in both tissue and organoids (51 in total) are labeled. (B) Expression levels of selected common DEGs plotted as individual data points in organoids as well as primary endometrial tissue; the mean±s.e.m. is shown. For primary tissue: CTRL, unaffected women; MRKH, MRKH patients. *n*=25 for CTRL, *n*=19 for MRKH.

Similarity between MRKH organoids and the previously analyzed endometrial tissue also existed with respect to DEGs. Comparing the set of 492 DEGs identified in MRKH organoids to the set of 2121 DEGs previously reported for MRKH endometrial tissue (Hentrich et al., 2020) led to 86 shared genes, of which 51 were dysregulated similarily in both organoid and tissue (Fig. 4A). Among them were the *GATA5* transcription factor as well as the *HOXB4* and *HOXB6* homeodomain proteins, which were upregulated in both MRKH tissue and organoid (Fig. 4B).

The relatively small overlap of DEGs between diseased tissue and organoid might partially be attributable to differences in tissue composition. Of the 2121 DEGs originating from the mixture of cell types (stromal, epithelial, endothelial and blood) in primary patient tissue (Hentrich et al., 2020), about one-third showed virtually no expression in organoids based on epithelial cells alone. Nevertheless, the overlapping DEGs that do exist suggest that the organoids capture parts of the pathology in a highly homogeneous and reproducible way.

### Widespread transcriptomic response of endometrial organoids to hormonal treatments

Next, we sought to investigate transcriptional changes upon treatment with steroid hormones in endometrial organoids of patients and controls, as the human endometrium undergoes substantial remodeling mainly controlled by the steroid hormones E2 and P4 during the menstrual cycle (Aghajanova et al., 2008; Roy and Matzuk, 2011). In order to investigate the hormone response of disease versus control organoids, RNA-sequencing data of E2- and E2+P4-treated organoids were analyzed for gene expression changes.

**Fig. 5.**
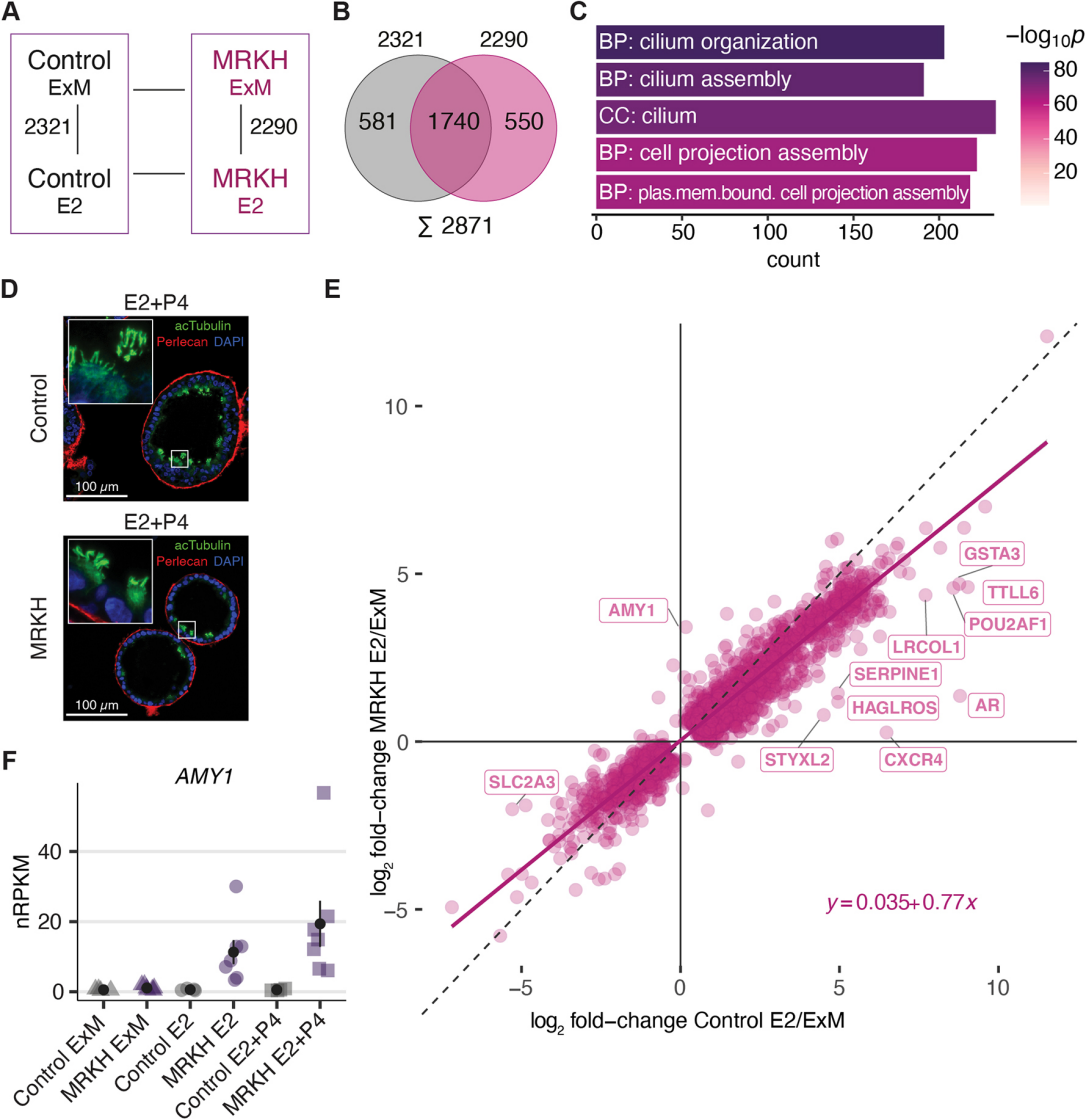
**Widespread transcriptomic response of organoids upon hormonal treatment with beta-estradiol.** (A) Diagram of experimental groups kept in expansion medium (ExM) or treated with beta-estradiol (E2). Numbers of differentially expressed genes are indicated for pairwise comparisons in control and MRKH organoids. (B) Venn diagram comparing common and distinct DEGs upon beta-estradiol treatment between MRKH (pink) and control (gray) organoids. (C) Enrichment analysis of overrepresented Gene Ontology terms among 2871 DEGs (total DEGs from the E2 and ExM conditions in MRKH and control organoids). Top five terms with the number of associated genes are shown according to their significance. (D) Representative immunofluorescence staining of a hormone-stimulated control (top) as well as a MRKH (bottom) organoid showing ciliated cells [green, indicated by acetylated α-tubulin (acTubulin)]. Perlecan staining (red) was used to represent the epithelial polarity, nuclei shown with DAPI (blue). Scale bars: 100 µm. Images are representative of at least 100 organoids (*n*=3). (E) Scatter plot of 2871 DEGs (total DEGs from the E2 and ExM conditions in MRKH and control organoids) comparing expression changes in control (*x*-axis) versus MRKH (*y*-axis) organoids. DEGs that differ in their altered expression by more than |log_2_ FC|>1 between control and MRKH organoids are labeled. (F) Expression levels of *AMY1* plotted as individual data points; the mean±s.e.m. is shown. For each treatment, *n*=4 for control, *n*=7 for MRKH.

Under E2 treatment, 2321 DEGs were identified in control and 2290 DEGs in MRKH organoids (Fig. 5A), of which about three-quarters overlapped (Fig. 5B). Furthermore, 594 of the DEGs have been described previously for endometrial epithelial organoids treated with E2 (Fitzgerald et al., 2019). As already indicated by the PCA (Fig. S2), the expression changes were highly homogenous between samples (Fig. S6A) and encompassed genes such as *FOXJ1*, which activates essential genes for motile cilia formation and function, as well as *DYDC2*, a marker for ciliated cells (Fig. S6B). Consistently, the most significant GO terms for these DEGs were ‘cilium organization’ and ‘cilium assembly’ (Fig. 5C); a fact that was also observed by immunostaining of hormone-treated organoids with the cilia marker acetylated alpha-tubulin (Fig. 5D). In addition, the expression of genes attributed to ciliated cells strongly increased upon treatment with E2 in both MRKH and control organoids (Figs S3 and S4).

Intriguingly, despite largely similar gene expression changes upon E2 treatment between MRKH and control organoids, a few genes were specific to disease condition (Fig. 5E). Among them were genes such as *AMY1* (also known as *AMY1A*) with upregulation upon E2 treatment specifically in MRKH organoids (Fig. 5F). Consistently and previously unnoticed, *AMY1* also showed increased expression in MRKH endometrial tissue (Fig. S6C). Hence, amylases – typically not expressed in endometrial tissue – might have been spuriously upregulated in MRKH patients, leading to tissue breakdown and degeneration.

As the next step, we studied the response of MRKH and control organoids to the combination of E2 and P4. As mentioned above, the DEG count was very similar and symmetric between E2 alone and the combination of E2 and P4 (Fig. S7). In fact, for both control (Fig. S7A) and MRKH organoids (Fig. S7B), gene expression changes under the hormone treatments were almost identical as reflected by the nearly perfect standard diagonal (Fig. S7C,D), and very few genes showed deviations (Fig. S7E), indicating that the addition of P4 to E2 led to few additional transcriptome changes.

### Validation of disease- and patient-specific gene expression changes in endometrial organoids

**Fig. 6.**
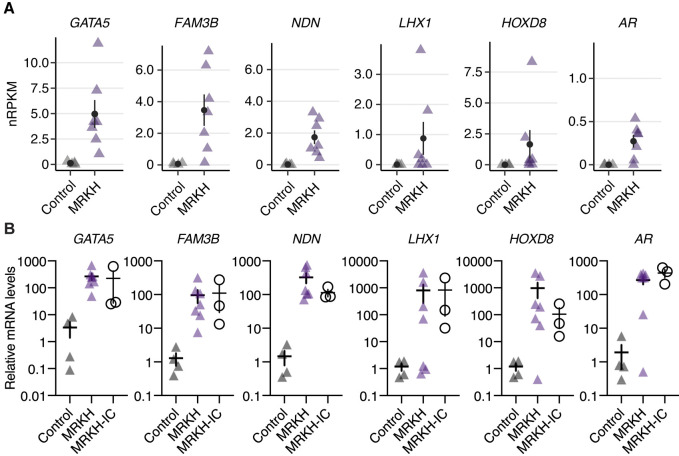
**Validation of selected condition- and patient-specific gene expression changes.** (A) Expression levels of *GATA5*, *FAM3B*, *NDN*, *LHX1*, *HOXD8* and *AR* plotted as individual data points based on RNA-sequencing data; the mean±s.e.m. is shown. *n*=4 for control, *n*=7 for MRKH. (B) Validation of expression changes seen in RNA sequencing of the sequencing cohort (control and MRKH; *n*=4 for control, *n*=7 for MRKH, respectively) as well as an independent cohort (MRKH-IC) consisting of three models. Expression levels of *GATA5*, *FAM3B*, *NDN*, *LHX1*, *HOXD8* and *AR* were investigated by RT-qPCR, using *SDHA* and *RPL13A* as reference genes. Data were normalized to control organoids to obtain relative mRNA levels and are shown as mean±s.e.m. Each triangle or circle represents an individual organoid line.

Finally, we validated disease- and patient-specific gene expression changes of the selected genes using quantitative PCR in the RNA-sequencing cohort as well as in an independent cohort (Fig. 6A). We focused on the DEGs that are also highly expressed: *LHX1* (associated in Müllerian agenesis; Huang et al., 2014), *HOXD8* (homeobox gene that is highly expressed during the development of the chicken MD; Roly et al., 2020), *FAM3B* (a recently identified FGFR ligand implicated in posterior development; Zhang et al., 2021), *NDN*, androgen receptor (*AR*) and *GATA5* (expressed during the MD development and associated with abnormalities of the genitourinary tract of female mice; Roly et al., 2020; Molkentin et al., 2000). Quantitative PCR assays clearly validated the RNA-sequencing results (Fig. 6B). Additionally, the expression changes found in our MRKH sequencing cohort were also found in the three MRKH models that were selected as an independent cohort (MRKH-IC) (Fig. 6B).

## DISCUSSION

During the last decade, a plethora of research projects, mainly in the field of genetics, have aimed at deciphering the cause of the multi-faceted MRKH syndrome (Herlin et al., 2020; Fontana et al., 2017). Reports of familial cases alongside seemingly sporadic appearances have fueled hope to find a genetic cause of the disease. Yet, a common genetic determinant has remained elusive thus far. Sequencing studies are mainly performed on blood samples and the focus lays primarily on mutations in the patients' germline. That, however, negates the possibility that the origin of MRKH syndrome lies in the developing tissue. MD development (Santana Gonzalez et al., 2021) is, as are all developmental processes, a highly complex sequence of events that involves activation and silencing of signaling pathways, expression of transcription factors (e.g. homeobox proteins) and cascades of growth factors, all of which must be precisely coordinated in space and time to arrive at the desired outcome (Mammoto and Ingber, 2010). Disturbances in these pathways either by lacking factors or their deregulated expressions may, hence, lead to developmental defects, as seen in MRKH patients with uterus aplasia alone (MRKH Type I) or with associated malformations (MRKH Type II).

### MRKH organoids show no evidence of impaired hormone receptor function

To find potential disease-causing alterations, we strongly advocate for focusing on diseased tissue, rather than sequencing patient blood. So far, only one study reports the successful culturing of endometrial stromal cells isolated from MRKH rudimentary uterine horns (Brucker et al., 2017). Here, we were able to isolate the endometrium from rudimentary uterine horns and establish long-term proliferating epithelial organoid cultures that share high similarity with healthy controls and are fully hormone responsive (Figs 2 and 5). This is in stark contrast to the previously isolated endometrial stromal cells that showed impaired decidualization upon steroid hormone exposure (Brucker et al., 2017). We hypothesize that these results point to a pivotal interplay between stromal and epithelial cells. *In vivo*, estrogen-stimulated stromal cells secrete growth factors (e.g. EGF and FGF-10) that lead to the proliferation of epithelial cells (Chung et al., 2015). Hence, impairment of estrogen receptivity in the stromal compartment might, in turn, lead to a reduction of these factors in MRKH patients and result in the reduced proliferation capacity seen *in vivo*, but not in the organoid model due to the supplementation of these factors in the growth medium (Table S2). Successful establishment and proliferation of endometrial cultures show that these cells are intrinsically able to proliferate once they are provided with external growth signals. These results suggest an essential crosstalk and interplay between stromal and epithelial cells in the pathogenesis of MRKH. The organoid model established here lends itself to further explore this theory and to continue to explore the causal factors of the disease.

### Female reproductive tract development

During development of the female reproductive tract, a cascade of tightly regulated processes in the developing embryo need to take place. This is largely governed by tightly controlled transcription factor programs, including homeobox-domain proteins. Clinically, MRKH represents a heterogeneous disease and is generally categorized into Type I (isolated finding) or Type II (accompanied by abnormalities of additional organ systems, primarily including the kidneys and the skeleton) (Herlin et al., 2020). Interestingly, in our patient cohort, about 90% of MRKH Type I cases had rudimentary horns, in contrast to Type II cases with only 38% (Table S1). The multifaceted appearance of the uterine horns (absent, one versus two, with endometrium or without and size variation) might point to defects at different phases of uterine development.

The uterus, both fallopian tubes, cervix and the upper third of the vagina develop from the MDs. This development can be divided into three phases (Santana Gonzalez et al., 2021). The first phase is the specification phase when Müllerian precursor cells are specified in the surface epithelium at the cranial pole of the mesonephros (embryonic kidney). During the second phase, termed invagination, these cells start to become mesoepithelial and move between the mesenchyme in a caudal direction. In the final phase, the elongation phase, invaginating cells from the MD epithelium contact the WD and the duct elongates. At this point, the most caudal part of the MD fuses into a single tube with a separating midline epithelial septum, forming the midline uterovaginal canal between the two adjacent MDs, which later disappears. The degree of midline fusion is vastly different between species. In mice, most of the MDs remain unfused and later give rise to large bilateral uterine horns instead of a single uterus in humans (Spencer et al., 2005). At this stage of development, the embryo is sexually indifferent and presents with both WDs and MDs (Kobayashi and Behringer, 2003). Under the influence of several signaling cascades and factors (e.g. AMH), the MDs regress in males, whereas lack of these hormones in females leads to regression of the WDs (Kobayashi and Behringer, 2003). After female sex determination, expression of Hox genes and retinoic acid (RA) signaling drive segmentation of the proximal and distal MD (Santana Gonzalez et al., 2021). Each specified Müllerian segment will give rise to a different part of the female reproductive tract, with the most anterior differentiating into the oviducts, and the most posterior into the upper vagina. We hypothesize that there is not a single defect in this finely tuned developmental process that causes MRKH syndrome, and that the multifaceted clinical phenotype can be attributed to the fact that different pathways were altered in different subsets of patients. MRKH patients without rudiments and patients with two developed rudiments containing endometrium were very likely to have different alterations in their genome and/or epigenome. The latter seemed to have had a caudal MD fusion defect whereas the former phenotype probably arose from a defect during the elongation phase. The fact that MRKH patients have fallopian tubes (Herlin et al., 2020), both emerging from MDs, provides evidence that the initial steps of MD development are still unchanged. Another possibility might be that the regression of the WDs (driven by apoptosis and degradation of tissue, which in turn are driven by metalloproteinases; Page-McCaw et al., 2007) in MRKH patients malfunctioned and led to partial regression of the adjacent MDs, which in turn led to a fusion defect in the uterovaginal canal. Hence, the structure regressed and could not establish a contact with the urogenital sinus that was forming the lower part of the vagina. Investigation of endometrial tissue of patients with other Müllerian anomalies such as bicornuate uterus, uterus didelphys (defect in fusion) or a uterus septum (defect in septal resorption) might provide important insights in the causality of MRKH as these likely represent similar, but milder defects, compared to MRKH patients.

### Role of the ECM and its interaction with the plasma membrane in MRKH

The ECM has been shown to play a pivotal role in various biological processes and is essential for the function and integrity of animal tissues (Lu et al., 2011). During development, the ECM is constantly being remodeled by degradation and reassembly processes. The female reproductive tract development, as outlined before, is marked by numerous such events of ECM remodeling. Interestingly, our RNA-sequencing results show an increase in the expression of genes involved in specific cellular processes and pathways related to the cellular compartments ‘plasma membrane’ and ‘cell periphery’, as well as ‘ECM-receptor interaction’, which were found to be highly enriched (Fig. 3C; Table S5). Although the GO terms ‘plasma membrane’ and ‘cell periphery’ are primarily only an indication of the location of the deregulated proteins, they suggest that the majority of DEGs in endometrial epithelial cells in MRKH organoids as compared to healthy controls are implicated in the interaction of glandular epithelial cells with the ECM and/or the stromal compartment of endometrium in the uterine rudiments. Moreover, when grown in culture, epithelial cells from MRKH patients have the potential to grow, respond to hormones and, in fact, behave very similarly to healthy organoids. This hints at the importance of the communication between epithelial and their surrounding cells for the normal development of the uterus, and highlights the importance of further investigating these interactions. Complex interactions between different cell types of the MRKH uterine rudiments have thus far not been implemented in the research community. Isolating stromal cells, as previously reported (Brucker et al., 2017), and performing co-culture experiments with endometrial organoid models might be a promising way of tackling this highly understudied research field. Furthermore, investigations using mouse models might be a promising tool to verify whether altered gene expression or knockouts of the candidate genes identified here might lead to genital malformations such as those seen for *LHX1* and other candidate genes (Herlin et al., 2020).

### Identification of disease-causing candidate genes in MRKH endometrial organoids

Uterine development occurs during the first trimester of development. At the time of surgery and tissue sampling, our patients have passed this point on average by 20 years (with one patient already being 41 years old) (Table S1). It is remarkable that we were able to establish fully functional endometrial organoids from this tissue, and even more remarkable that we were able to find specific expression differences in 492 DEGs including already suspected candidate genes such as *LHX1* and Hox genes. Surprisingly, these were mainly found to be expressed in MRKH organoids and absent in healthy controls. We speculate that this explains why any single candidate gene may not necessarily be mutated in all patients. Mutations that cause a complete loss of function might have very severe developmental defects leading to embryonic lethality and, hence, cannot be discovered. For example, homozygous *Lhx1* knockout in mice leads to only a few neonates that are born with an MRKH-like phenotype, but also lack anterior head structures (Kobayashi et al., 2004). A deregulation (but not necessarily a knockout) of this gene, specifically during uterine development or in specific regions of the developing female reproductive tract, might however cause MD anomalies while leaving other regions unharmed. The fact that a large portion of the MRKH candidate gene identification data is curated from knockout studies in animal models (Masse et al., 2009) might be the reason why many of our identified genes have not yet been associated with the disease. The transcription factor GATA5, for example, is highly expressed in MRKH endometrial organoids as well as in tissue (Hentrich et al., 2020) compared to healthy controls, but has never been linked with MD anomalies, even though it is highly and dynamically expressed in the developing tissue, as revealed by a large-scale RNA-sequencing study in chicken (Roly et al., 2020). In the future, shifting the focus from knockout studies towards interrogation of dysregulated gene expression using CRISPR-A screens (Kampmann, 2018) might help to better understand the processes during uterine development. Several of our identified DEGs could serve as a starting point to select interesting candidates.

### The importance of identifying the cause of MRKH syndrome

MRKH syndrome is the most common cause of uterine aplasia at a frequency estimated to be 1 in 4500 female newborns. Since the early 2000s, treatment of MRKH patients mainly focuses on the co-existing vaginal aplasia by creation of a neovagina to give patients the possibility of sexual intercourse (Rall et al., 2014). This vastly improves the quality of life of affected women (Weijenborg et al., 2019). However, this treatment is not adequate to provide a solution for the missing reproductive ability in MRKH patients. Besides adoption and surrogacy, uterus transplantation has emerged as a highly promising method to overcome this shortcoming (Brannstrom et al., 2018). The two latter methods come with a plethora of ethical difficulties, and researchers and clinicians are working urgently to find new ways of providing larger groups of patients with a cure. Organ-on-a-chip-based methods as well as using animals or deceased patient uterus scaffolds represent opportunities for uteri reconstruction using patient-specific cells to overcome limitations of transplantation approaches (Bergmann et al., 2021). The development of these techniques in combination with an in-depth understanding of uterine development might mark a milestone for achieving a long-term treatment solution to some of the most severe uterine pathologies such as MRKH syndrome. To use patient-derived cells for uterus reconstruction, we need to get a deeper understanding of their functionality and their interactions. Hence, endometrial organoids from MRKH patients and their interaction with stromal cells need to be investigated further to achieve this goal.

Our study shows that MRKH endometrial organoids are hormone responsive and show a high similarity to healthy endometrial epithelial cells. Future research in this emerging line of research, however, should reveal whether this is sufficient for successful embryo implantation in a to-be-developed *de novo* uterus from MRKH patient cells (Alzamil et al., 2021; Bergmann et al., 2021).

## MATERIALS AND METHODS

### Patient cohort

All tissue biopsies were obtained from patients after informed written consent. The study was conducted according to the guidelines of the Declaration of Helsinki and was approved by the Ethics Committee of the Eberhard Karl University of Tübingen (Ethical approval 205/2014BO1 and 150/2018BO2) and is compliant with all relevant ethical regulations regarding research involving human participants. Rudimentary uterine horns were excised from 37 MRKH patients at the time of laparoscopically assisted creation of a neovagina and transported to the pathology lab in sterile containers. Uterine rudiments were sectioned perpendicular to the longest axis and macroscopically evaluated for presence of endometrial tissue. If present, endometrium was removed with as little attached myometrium as possible and further processed. For this study, endometrial tissue from 12 patients with MRKH syndrome was collected. Endometrial biopsy samples [obtained via Pipelle Endometrial Suction Curette (Medesign IC)] of four premenopausal patients served as controls. For full patient characteristics, see Table S1.

### Processing of endometrium samples and organoid culture setup

Tissue samples were minced into small pieces (1–3 mm^3^) using a scalpel and dissociated with collagenase/dispase (1 mg/ml; COLLDISP-RO, Roche) in the presence of Rock inhibitor (Y-27632, 10 μM; M1817, Abmole Bioscience) for 1 h at 37°C on a shaking table. The digestion was attenuated by addition of medium [advanced Dulbecco's modified Eagle medium (DMEM)/F12 (12634010, Gibco) without serum] and centrifuged at 478 ***g*** for 10 min. The final pellet was resuspended in advanced DMEM/F12 supplemented with 1% Glutamax, 1% HEPES and 1% penicillin/streptomycin (all Gibco) and the desired amount of cell suspension mixed with BME (Type II, 3533-001-2, Trevigen) at a ratio of 65% BME to 35% cell suspension. 20 µl droplets were plated out on pre-warmed 48-well plates and placed upside-down in an incubator (37°C, 5% CO_2_) for solidification. Afterwards, culture medium (Table S2) was added to each well and renewed every 3 days. Noggin conditioned medium from HEK293T-Noggin-Fc-cells (kindly provided by Hans Clevers, Hubrecht Institute, Utrecht, Netherlands) was produced as previously described (Farin et al., 2012). R-Spondin conditioned medium was produced with Cultrex^®^ HA-R-Spondin1-Fc 293T Cells (Trevigen) according to the distributor's protocol.

### Passaging/cryopreservation of organoid cultures

For passaging or cryopreservation, organoids were recovered by resuspending the BME drops in ice-cold advanced DMEM/F12 and transferred to 15 ml tubes. The organoid suspension was either mechanically or enzymatically [25% 1× TrypLE Express (Gibco), 75% advanced DMEM/F12] dispersed and then pelleted. For further culture, the pellet was reconstituted in advanced DMEM/F12 and mixed with BME at a ratio of 65% BME to 35% cell suspension and cultured as described above. For cryopreservation, the cell pellet was resuspended in Recovery™ Cell Culture Freezing Medium (Gibco), the solution transferred to cryo-vials and then cooled down in CoolCell™ LX Freezing Containers (Merck) in a −80°C freezer. The next day, vials were transferred for long-term storage to liquid nitrogen tanks.

### Paraffin sections and immunohistochemistry

Tissues and organoids were fixed in 4% paraformaldehyde followed by dehydration, paraffin embedding, sectioning (4 µm) and standard Haematoxylin and Eosin staining. Immunohistochemistry was performed on a Ventana Discovery automated immunostaining system (Ventana Medical Systems, Tucson, AZ, USA) using antibodies as specified in Table S3.

### Immunofluorescence

For characterization of organoids, formalin-fixed paraffin-embedded (FFPE) sections (4 µm) were subjected to heat-induced antigen retrieval and incubated with primary antibodies as specified in Table S3. Sections were incubated overnight in a humidified chamber at 4°C, and afterwards washed three times with PBS containing 0.1% Tween-20 and incubated for 1 h at room temperature with the respective secondary antibody (Table S3). Finally, the sections were again washed and mounted in ProLong Diamond Antifade mounting media containing DAPI (Thermo Fisher Scientific). Images were acquired with the EVOS M7000 imaging system and processed using ImageJ (NIH, USA).

### Hormone treatment of organoid cultures

Organoids were passaged as described above and allowed to grow for 4 days in standard culture medium (expansion medium; ExM). Afterwards, three different groups were established. Group 1 (ExM) served as an untreated control sample and received ExM for an additional 6 days, with the medium refreshed every other day. Group 2 (E2) was cultured with ExM containing 10 nM beta-estradiol (E8875, Sigma) for 6 days. Again, the medium was renewed every other day. Group 3 (E2+P4) first received ExM supplemented with 10 nM beta-estradiol for 2 days and afterwards ExM with 1 µM progesterone (P8783, Sigma) and 1 µM cAMP (1140, Tocris) in addition to 10 nM beta-estradiol for additional 4 days. After a total of 10 days, all organoids were harvested. For this, medium was removed from the wells and the BME domes incubated with 1× TrypLE Express at 37°C for 10 min. PBS was added to dilute the TrypLE Express and the suspension centrifuged at 478 ***g*** for 10 min. After discarding the supernatant, the pellet was resuspended in PBS and centrifuged again to remove leftover BME. After removing the supernatant, cell pellets were snap frozen in liquid nitrogen and then stored at −80°C.

### RNA isolation and sequencing

Total RNA was isolated from organoid cultures using the RNeasy Mini Kit (74004, Qiagen). Simultaneous elimination of genomic DNA was achieved with on-column DNA digestion (79254, Qiagen) according to the manufacturer's protocol. The quality was assessed with an Agilent 2100 Bioanalyzer. Using the NEBNext Ultra II Directional RNA Library Prep Kit for Illumina and 100 ng of total RNA for each sequencing library, poly(A)-selected paired-end sequencing libraries (101 bp read length) were generated according to the manufacturer's instructions. All libraries were sequenced on an Illumina NovaSeq 6000 platform at a depth of around 40 million reads each. Library preparation and sequencing procedures were performed by the same individual, and a design aimed to minimize technical batch effects was chosen.

### Quality control, alignment and differential expression analysis

Read quality of RNA-seq data in fastq files was assessed using FastQC (v0.11.9) (https://www.bioinformatics.babraham.ac.uk/projects/fastqc/) to identify sequencing cycles with low average quality, adaptor contamination or repetitive sequences from PCR amplification. Reads were aligned using *STAR* (v2.7.6a) (Dobin et al., 2013) allowing gapped alignments to account for splicing against the *Homo sapiens* genome from GENCODE v35. Alignment quality was analyzed using SAMtools (v1.10) (Li et al., 2009). Normalized read counts for all genes were obtained using DESeq2 (v1.32.0) (Love et al., 2014). Transcripts covered with less than 50 reads (median of all samples) were excluded from the analysis, leaving 15,413 genes for determining differential expression. Cut-offs of |log_2_FC|≥0.5 and *P*_adj_≤0.05 were set to determine differentially expressed genes. Gene-level abundances were derived from DESeq2 as normalized read counts, and used for calculating the log_2_-transformed expression changes underlying the expression heatmaps, for which ratios were computed against mean expression in control samples. The normalized counts provided by the ‘sizeFactors’ function in DESeq2 also went into calculating normalized reads per kilobase per million total reads (nRPKMs) as a measure of relative gene expression (Srinivasan et al., 2016).

### Gene annotation, enrichment and regulator analyses

g:Profiler2 (v0.2.0) was employed to identify overrepresented GO terms for differentially expressed genes (Raudvere et al., 2019). Transcription factor-binding site analyses were carried out in Pscan (v1.6) (Zambelli et al., 2009) on the *Homo sapiens* genome considering the −450 to +50 bp of the promoter region for motifs against the Jaspar 2020_NR database. TFEA.ChIP (v1.12.0) was employed with default parameters to determine transcription factor enrichment using the initial database version of ChIP-seq experiments (Puente-Santamaria et al., 2019). Cell-type-specific endometrial marker genes were taken from a study with single-cell profiles from uterine tissue (Wang et al., 2020), as well as endometrial epithelial organoids (Fitzgerald et al., 2019).

### Reverse transcription quantitative PCR (RT-qPCR)

Total RNA was extracted from organoids using the RNeasy Mini Kit (74004, Qiagen), simultaneously eliminating genomic DNA with on-column DNA digestion (79254, Qiagen). Equal amounts of total RNA (1 μg) were reverse transcribed using the Maxima™ H Minus cDNA Synthesis Master Mix (M1661, Thermo Fisher Scientific). To analyze gene expression, 5 ng cDNA was subjected to real-time qPCR using PowerUp™ SYBR^®^ Green Mastermix (A25741, Thermo Fisher Scientific) on the QuantStudio 3 Real-Time PCR system (A28567, Thermo Fisher Scientific). Thermal cycling was performed with 3 min at 95°C, followed by 40 cycles of 95°C for 15 s and 60°C for 60 s. The specificity of the RT-qPCR products was assessed by melting curve analysis. Relative quantification was performed using the 2^−ΔΔCt^ method with *SDHA* and *RPL13A* as reference genes. Expression was normalized to the endometrial control group. All experiments were performed in duplicates. PCR primers are listed in Table S4.

## Supplementary Material

Supplementary information

## References

[DMM049379C1] Aghajanova, L., Hamilton, A. E. and Giudice, L. C. (2008). Uterine receptivity to human embryonic implantation: histology, biomarkers, and transcriptomics. *Semin. Cell Dev. Biol.* 19, 204-211. 10.1016/j.semcdb.2007.10.008 18035563 PMC2829661

[DMM049379C2] Aittomäki, K., Eroila, H. and Kajanoja, P. (2001). A population-based study of the incidence of müllerian aplasia in Finland. *Fertil. Steril.* 76, 624-625. 10.1016/S0015-0282(01)01963-X 11570363

[DMM049379C3] Alzamil, L., Nikolakopoulou, K. and Turco, M. Y. (2021). Organoid systems to study the human female reproductive tract and pregnancy. *Cell Death Differ.* 28, 35-51. 10.1038/s41418-020-0565-5 32494027 PMC7852529

[DMM049379C5] Bergmann, S., Schindler, M., Munger, C., Penfold, C. A. and Boroviak, T. E. (2021). Building a stem cell-based primate uterus. *Commun Biol* 4, 749. 10.1038/s42003-021-02233-8 34140619 PMC8211708

[DMM049379C6] Biason-Lauber, A., Konrad, D., Navratil, F. and Schoenle, E. J. (2004). A WNT4 mutation associated with Müllerian-duct regression and virilization in a 46,XX woman. *N. Engl. J. Med.* 351, 792-798. 10.1056/NEJMoa040533 15317892

[DMM049379C7] Biason-Lauber, A., DE Filippo, G., Konrad, D., Scarano, G., Nazzaro, A. and Schoenle, E. J. (2007). WNT4 deficiency--a clinical phenotype distinct from the classic Mayer-Rokitansky-Kuster-Hauser syndrome: a case report. *Hum. Reprod.* 22, 224-229. 10.1093/humrep/del360 16959810

[DMM049379C8] Boretto, M., Cox, B., Noben, M., Hendriks, N., Fassbender, A., Roose, H., Amant, F., Timmerman, D., Tomassetti, C., Vanhie, A. et al. (2017). Development of organoids from mouse and human endometrium showing endometrial epithelium physiology and long-term expandability. *Development* 144, 1775-1786. 10.1242/dev.148478 28442471

[DMM049379C9] Boretto, M., Maenhoudt, N., Luo, X., Hennes, A., Boeckx, B., Bui, B., Heremans, R., Perneel, L., Kobayashi, H., VAN Zundert, I. et al. (2019). Patient-derived organoids from endometrial disease capture clinical heterogeneity and are amenable to drug screening. *Nat. Cell Biol.* 21, 1041-1051. 10.1038/s41556-019-0360-z 31371824

[DMM049379C10] Brannstrom, M., Dahm Kähler, P., Greite, R., Molne, J., Díaz-García, C. and Tullius, S. G. (2018). Uterus transplantation: a rapidly expanding field. *Transplantation* 102, 569-577. 10.1097/TP.0000000000002035 29210893

[DMM049379C11] Brucker, S. Y., Eisenbeis, S., König, J., Lamy, M., Salker, M. S., Zeng, N., Seeger, H., Henes, M., Schöller, D., Schönfisch, B. et al. (2017). Decidualization is Impaired in Endometrial Stromal Cells from Uterine Rudiments in Mayer-Rokitansky-Kuster-Hauser Syndrome. *Cell. Physiol. Biochem.* 41, 1083-1097. 10.1159/000464116 28245469

[DMM049379C12] Chung, D., Gao, F., Jegga, A. G. and Das, S. K. (2015). Estrogen mediated epithelial proliferation in the uterus is directed by stromal Fgf10 and Bmp8a. *Mol. Cell. Endocrinol.* 400, 48-60. 10.1016/j.mce.2014.11.002 25451979 PMC4751583

[DMM049379C13] Dobin, A., Davis, C. A., Schlesinger, F., Drenkow, J., Zaleski, C., Jha, S., Batut, P., Chaisson, M. and Gingeras, T. R. (2013). STAR: ultrafast universal RNA-seq aligner. *Bioinformatics* 29, 15-21. 10.1093/bioinformatics/bts635 23104886 PMC3530905

[DMM049379C14] Farin, H. F., van Es, J. H. and Clevers, H. (2012). Redundant sources of Wnt regulate intestinal stem cells and promote formation of Paneth cells. *Gastroenterology* 143, 1518-1529.e7. 10.1053/j.gastro.2012.08.031 22922422

[DMM049379C15] Fitzgerald, H. C., Dhakal, P., Behura, S. K., Schust, D. J. and Spencer, T. E. (2019). Self-renewing endometrial epithelial organoids of the human uterus. *Proc. Natl. Acad. Sci. USA* 116, 23132-23142. 10.1073/pnas.1915389116 31666317 PMC6859318

[DMM049379C16] Fontana, L., Gentilin, B., Fedele, L., Gervasini, C. and Miozzo, M. (2017). Genetics of Mayer-Rokitansky-Küster-Hauser (MRKH) syndrome. *Clin. Genet.* 91, 233-246. 10.1111/cge.12883 27716927

[DMM049379C17] Göllner, H., Bouwman, P., Mangold, M., Karis, A., Braun, H., Rohner, I., del Rey, A., Besedovsky, H. O., Meinhardt, A., van den Broek, M. et al. (2001). Complex phenotype of mice homozygous for a null mutation in the Sp4 transcription factor gene. *Genes Cells* 6, 689-697. 10.1046/j.1365-2443.2001.00455.x 11532028

[DMM049379C18] Hentrich, T., Koch, A., Weber, N., Kilzheimer, A., Maia, A., Burkhardt, S., Rall, K., Casadei, N., Kohlbacher, O., Riess, O. et al. (2020). The endometrial transcription landscape of MRKH Syndrome. *Front. Cell Dev. Biol.* 8, 572281. 10.3389/fcell.2020.572281 33072755 PMC7542331

[DMM049379C19] Herlin, M., Højland, A. T. and Petersen, M. B. (2014). Familial occurrence of Mayer-Rokitansky-Küster-Hauser syndrome: a case report and review of the literature. *Am. J. Med. Genet. A* 164, 2276-2286. 10.1002/ajmg.a.36652 24975471

[DMM049379C20] Herlin, M., Bjørn, A.-M. B., Rasmussen, M., Trolle, B. and Petersen, M. B. (2016). Prevalence and patient characteristics of Mayer-Rokitansky-Kuster-Hauser syndrome: a nationwide registry-based study. *Hum. Reprod.* 31, 2384-2390. 10.1093/humrep/dew220 27609979

[DMM049379C21] Herlin, M. K., Petersen, M. B. and Brännström, M. (2020). Mayer-Rokitansky-Kuster-Hauser (MRKH) syndrome: a comprehensive update. *Orphanet J. Rare Dis.* 15, 214. 10.1186/s13023-020-01491-9 32819397 PMC7439721

[DMM049379C22] Huang, C.-C., Orvis, G. D., Kwan, K. M. and Behringer, R. R. (2014). Lhx1 is required in Müllerian duct epithelium for uterine development. *Dev. Biol.* 389, 124-136. 10.1016/j.ydbio.2014.01.025 24560999 PMC3988469

[DMM049379C23] Kampmann, M. (2018). CRISPRi and CRISPRa screens in mammalian cells for precision biology and medicine. *ACS Chem. Biol.* 13, 406-416. 10.1021/acschembio.7b00657 29035510 PMC5886776

[DMM049379C24] Kobayashi, A. and Behringer, R. R. (2003). Developmental genetics of the female reproductive tract in mammals. *Nat. Rev. Genet.* 4, 969-980. 10.1038/nrg1225 14631357

[DMM049379C25] Kobayashi, A., Shawlot, W., Kania, A. and Behringer, R. R. (2004). Requirement of Lim1 for female reproductive tract development. *Development* 131, 539-549. 10.1242/dev.00951 14695376

[DMM049379C26] Ledig, S., Schippert, C., Strick, R., Beckmann, M. W., Oppelt, P. G. and Wieacker, P. (2011). Recurrent aberrations identified by array-CGH in patients with Mayer-Rokitansky-Küster-Hauser syndrome. *Fertil. Steril.* 95, 1589-1594. 10.1016/j.fertnstert.2010.07.1062 20797712

[DMM049379C27] Ledig, S., Brucker, S., Barresi, G., Schomburg, J., Rall, K. and Wieacker, P. (2012). Frame shift mutation of LHX1 is associated with Mayer-Rokitansky-Kuster-Hauser (MRKH) syndrome. *Hum. Reprod.* 27, 2872-2875. 10.1093/humrep/des206 22740494

[DMM049379C28] Li, H., Handsaker, B., Wysoker, A., Fennell, T., Ruan, J., Homer, N., Marth, G., Abecasis, G., Durbin, R. and 1000 Genome Project Data Processing Subgroup. (2009). The Sequence Alignment/Map format and SAMtools. *Bioinformatics* 25, 2078-2079. 10.1093/bioinformatics/btp352 19505943 PMC2723002

[DMM049379C29] Love, M. I., Huber, W. and Anders, S. (2014). Moderated estimation of fold change and dispersion for RNA-seq data with DESeq2. *Genome Biol.* 15, 550. 10.1186/s13059-014-0550-8 25516281 PMC4302049

[DMM049379C30] Lu, P., Takai, K., Weaver, V. M. and Werb, Z. (2011). Extracellular matrix degradation and remodeling in development and disease. *Cold Spring Harb. Perspect. Biol.* 3, a005058. 10.1101/cshperspect.a005058 21917992 PMC3225943

[DMM049379C31] Mammoto, T. and Ingber, D. E. (2010). Mechanical control of tissue and organ development. *Development* 137, 1407-1420. 10.1242/dev.024166 20388652 PMC2853843

[DMM049379C32] Masse, J., Watrin, T., Laurent, A., Deschamps, S., Guerrier, D. and Pellerin, I. (2009). The developing female genital tract: from genetics to epigenetics. *Int. J. Dev. Biol.* 53, 411-424. 10.1387/ijdb.082680jm 19412895

[DMM049379C33] Miyamoto, N., Yoshida, M., Kuratani, S., Matsuo, I. and Aizawa, S. (1997). Defects of urogenital development in mice lacking Emx2. *Development* 124, 1653-1664. 10.1242/dev.124.9.1653 9165114

[DMM049379C34] Molkentin, J. D., Tymitz, K. M., Richardson, J. A. and Olson, E. N. (2000). Abnormalities of the genitourinary tract in female mice lacking GATA5. *Mol. Cell. Biol.* 20, 5256-5260. 10.1128/MCB.20.14.5256-5260.2000 10866681 PMC85974

[DMM049379C35] Mullen, R. D. and Behringer, R. R. (2014). Molecular genetics of Müllerian duct formation, regression and differentiation. *Sex Dev* 8, 281-296. 10.1159/000364935 25033758 PMC4378544

[DMM049379C36] Nik-Zainal, S., Strick, R., Storer, M., Huang, N., Rad, R., Willatt, L., Fitzgerald, T., Martin, V., Sandford, R., Carter, N. P. et al. (2011). High incidence of recurrent copy number variants in patients with isolated and syndromic Müllerian aplasia. *J. Med. Genet.* 48, 197-204. 10.1136/jmg.2010.082412 21278390 PMC3322361

[DMM049379C37] Oppelt, P., Renner, S. P., Kellermann, A., Brucker, S., Hauser, G. A., Ludwig, K. S., Strissel, P. L., Strick, R., Wallwiener, D. and Beckmann, M. W. (2006). Clinical aspects of Mayer-Rokitansky-Kuester-Hauser syndrome: recommendations for clinical diagnosis and staging. *Hum. Reprod.* 21, 792-797. 10.1093/humrep/dei381 16284062

[DMM049379C38] Oppelt, P. G., Lermann, J., Strick, R., Dittrich, R., Strissel, P., Rettig, I., Schulze, C., Renner, S. P., Beckmann, M. W., Brucker, S. et al. (2012). Malformations in a cohort of 284 women with Mayer-Rokitansky-Küster-Hauser syndrome (MRKH). *Reprod. Biol. Endocrinol.* 10, 57. 10.1186/1477-7827-10-57 22906151 PMC3489887

[DMM049379C39] Page-Mccaw, A., Ewald, A. J. and Werb, Z. (2007). Matrix metalloproteinases and the regulation of tissue remodelling. *Nat. Rev. Mol. Cell Biol.* 8, 221-233. 10.1038/nrm2125 17318226 PMC2760082

[DMM049379C40] Philibert, P., Biason-Lauber, A., Rouzier, R., Pienkowski, C., Paris, F., Konrad, D., Schoenle, E. and Sultan, C. (2008). Identification and functional analysis of a new WNT4 gene mutation among 28 adolescent girls with primary amenorrhea and müllerian duct abnormalities: a French collaborative study. *J. Clin. Endocrinol. Metab.* 93, 895-900. 10.1210/jc.2007-2023 18182450

[DMM049379C41] Philibert, P., Biason-Lauber, A., Gueorguieva, I., Stuckens, C., Pienkowski, C., Lebon-Labich, B., Paris, F. and Sultan, C. (2011). Molecular analysis of WNT4 gene in four adolescent girls with mullerian duct abnormality and hyperandrogenism (atypical Mayer-Rokitansky-Kuster-Hauser syndrome). *Fertil. Steril.* 95, 2683-2686. 10.1016/j.fertnstert.2011.01.152 21377155

[DMM049379C42] Puente-Santamaria, L., Wasserman, W. W. and del Peso, L. (2019). TFEA.ChIP: a tool kit for transcription factor binding site enrichment analysis capitalizing on ChIP-seq datasets. *Bioinformatics*. 35, 5339-5340. 10.1093/bioinformatics/btz573 31347689

[DMM049379C43] Rall, K., Barresi, G., Wallwiener, D., Brucker, S. Y. and Staebler, A. (2013). Uterine rudiments in patients with Mayer-Rokitansky-Küster-Hauser syndrome consist of typical uterine tissue types with predominantly basalis-like endometrium. *Fertil. Steril.* 99, 1392-1399. 10.1016/j.fertnstert.2012.12.002 23321321

[DMM049379C44] Rall, K., Schickner, M. C., Barresi, G., Schönfisch, B., Wallwiener, M., Wallwiener, C. W., Wallwiener, D. and Brucker, S. Y. (2014). Laparoscopically assisted neovaginoplasty in vaginal agenesis: a long-term outcome study in 240 patients. *J. Pediatr. Adolesc. Gynecol.* 27, 379-385. 10.1016/j.jpag.2014.02.002 25256875

[DMM049379C45] Rall, K., Eisenbeis, S., Barresi, G., Ruckner, D., Walter, M., Poths, S., Wallwiener, D., Riess, O., Bonin, M. and Brucker, S. (2015a). Mayer-Rokitansky-Kuster-Hauser syndrome discordance in monozygotic twins: matrix metalloproteinase 14, low-density lipoprotein receptor-related protein 10, extracellular matrix, and neoangiogenesis genes identified as candidate genes in a tissue-specific mosaicism. *Fertil. Steril.* 103, 494-502.e3. 10.1016/j.fertnstert.2014.10.053 25492683

[DMM049379C46] Rall, K., Eisenbeis, S., Henninger, V., Henes, M., Wallwiener, D., Bonin, M. and Brucker, S. (2015b). Typical and atypical associated findings in a group of 346 patients with Mayer-Rokitansky-Kuester-Hauser Syndrome. *J. Pediatr. Adolesc. Gynecol.* 28, 362-368. 10.1016/j.jpag.2014.07.019 26148785

[DMM049379C47] Raudvere, U., Kolberg, L., Kuzmin, I., Arak, T., Adler, P., Peterson, H. and Vilo, J. (2019). g:Profiler: a web server for functional enrichment analysis and conversions of gene lists (2019 update). *Nucleic Acids Res.* 47, W191-W198. 10.1093/nar/gkz369 31066453 PMC6602461

[DMM049379C48] Robboy, S. J., Kurita, T., Baskin, L. and Cunha, G. R. (2017). New insights into human female reproductive tract development. *Differentiation* 97, 9-22. 10.1016/j.diff.2017.08.002 28918284 PMC5712241

[DMM049379C49] Roly, Z. Y., Backhouse, B., Cutting, A., Tan, T. Y., Sinclair, A. H., Ayers, K. L., Major, A. T. and Smith, C. A. (2018). The cell biology and molecular genetics of Mullerian duct development. *Wiley Interdiscip. Rev. Dev. Biol.* 7, e310. 10.1002/wdev.310 29350886

[DMM049379C50] Roly, Z. Y., Godini, R., Estermann, M. A., Major, A. T., Pocock, R. and Smith, C. A. (2020). Transcriptional landscape of the embryonic chicken Mullerian duct. *BMC Genomics* 21, 688. 10.1186/s12864-020-07106-8 33008304 PMC7532620

[DMM049379C51] Roy, A. and Matzuk, M. M. (2011). Reproductive tract function and dysfunction in women. *Nat. Rev. Endocrinol.* 7, 517-525. 10.1038/nrendo.2011.79 21610684

[DMM049379C52] Sandbacka, M., Laivuori, H., Freitas, E., Halttunen, M., Jokimaa, V., Morin-Papunen, L., Rosenberg, C. and Aittomaki, K. (2013). TBX6, LHX1 and copy number variations in the complex genetics of Mullerian aplasia. *Orphanet J. Rare Dis.* 8, 125. 10.1186/1750-1172-8-125 23954021 PMC3847609

[DMM049379C53] Santana Gonzalez, L., Rota, I. A., Artibani, M., Morotti, M., Hu, Z., Wietek, N., Alsaadi, A., Albukhari, A., Sauka-Spengler, T. and Ahmed, A. A. (2021). Mechanistic drivers of Müllerian duct development and differentiation into the oviduct. *Front. Cell Dev. Biol.* 9, 605301. 10.3389/fcell.2021.605301 33763415 PMC7982813

[DMM049379C54] Spencer, T. E., Hayashi, K., Hu, J. and Carpenter, K. D. (2005). Comparative developmental biology of the mammalian uterus. *Curr. Top. Dev. Biol.* 68, 85-122. 10.1016/S0070-2153(05)68004-0 16124997

[DMM049379C55] Srinivasan, K., Friedman, B. A., Larson, J. L., Lauffer, B. E., Goldstein, L. D., Appling, L. L., Borneo, J., Poon, C., Ho, T., Cai, F. et al. (2016). Untangling the brain's neuroinflammatory and neurodegenerative transcriptional responses. *Nat. Commun.* 7, 11295. 10.1038/ncomms11295 27097852 PMC4844685

[DMM049379C56] Tewes, A.-C., Rall, K. K., Romer, T., Hucke, J., Kapczuk, K., Brucker, S., Wieacker, P. and Ledig, S. (2015). Variations in RBM8A and TBX6 are associated with disorders of the Müllerian ducts. *Fertil. Steril.* 103, 1313-1318. 10.1016/j.fertnstert.2015.02.014 25813282

[DMM049379C57] Tewes, A.-C., Hucke, J., Römer, T., Kapczuk, K., Schippert, C., Hillemanns, P., Wieacker, P. and Ledig, S. (2019). Sequence variants in TBX6 are associated with disorders of the Mullerian ducts: an update. *Sex. Dev.* 13, 35-40. 10.1159/000496819 30739119

[DMM049379C58] Timmreck, L. S. and Reindollar, R. H. (2003). Contemporary issues in primary amenorrhea. *Obstet. Gynecol. Clin. North Am.* 30, 287-302. 10.1016/S0889-8545(03)00027-5 12836721

[DMM049379C59] Turco, M. Y., Gardner, L., Hughes, J., Cindrova-Davies, T., Gomez, M. J., Farrell, L., Hollinshead, M., Marsh, S. G. E., Brosens, J. J., Critchley, H. O. et al. (2017). Long-term, hormone-responsive organoid cultures of human endometrium in a chemically defined medium. *Nat. Cell Biol.* 19, 568-577. 10.1038/ncb3516 28394884 PMC5410172

[DMM049379C60] Vainio, S., Heikkilä, M., Kispert, A., Chin, N. and Mcmahon, A. P. (1999). Female development in mammals is regulated by Wnt-4 signalling. *Nature* 397, 405-409. 10.1038/17068 9989404

[DMM049379C61] Wang, M., Li, Y., Ma, W., Li, H., He, F., Pu, D., Su, T. and Wang, S. (2014). Analysis of WNT9B mutations in Chinese women with Mayer-Rokitansky-Küster-Hauser syndrome. *Reprod. Biomed. Online* 28, 80-85. 10.1016/j.rbmo.2013.09.022 24268733

[DMM049379C62] Wang, W., Vilella, F., Alama, P., Moreno, I., Mignardi, M., Isakova, A., Pan, W., Simon, C. and Quake, S. R. (2020). Single-cell transcriptomic atlas of the human endometrium during the menstrual cycle. *Nat. Med.* 26, 1644-1653. 10.1038/s41591-020-1040-z 32929266

[DMM049379C63] Waschk, D. E. J., Tewes, A.-C., Römer, T., Hucke, J., Kapczuk, K., Schippert, C., Hillemanns, P., Wieacker, P. and Ledig, S. (2016). Mutations in WNT9B are associated with Mayer-Rokitansky-Kuster-Hauser syndrome. *Clin. Genet.* 89, 590-596. 10.1111/cge.12701 26610373

[DMM049379C64] Weijenborg, P. T. M., Kluivers, K. B., Dessens, A. B., Kate-Booij, M. J. and Both, S. (2019). Sexual functioning, sexual esteem, genital self-image and psychological and relational functioning in women with Mayer-Rokitansky-Küster-Hauser syndrome: a case-control study. *Hum. Reprod.* 34, 1661-1673. 10.1093/humrep/dez130 31418785

[DMM049379C65] Williams, L. S., Demir Eksi, D., Shen, Y., Lossie, A. C., Chorich, L. P., Sullivan, M. E., Phillips, J. A., III, Erman, M., Kim, H. G., Alper, O. M. and et al. (2017). Genetic analysis of Mayer-Rokitansky-Kuster-Hauser syndrome in a large cohort of families. *Fertil. Steril.* 108, 145-151.e2. 10.1016/j.fertnstert.2017.05.017 28600106 PMC5770980

[DMM049379C66] Zambelli, F., Pesole, G. and Pavesi, G. (2009). Pscan: finding over-represented transcription factor binding site motifs in sequences from co-regulated or co-expressed genes. *Nucleic Acids Res.* 37, W247-W252. 10.1093/nar/gkp464 19487240 PMC2703934

[DMM049379C67] Zhang, F., Zhu, X., Wang, P., He, Q., Huang, H., Zheng, T., Li, Y., Jia, H., Xu, L., Zhao, H. et al. (2021). The cytokine FAM3B/PANDER is an FGFR ligand that promotes posterior development in Xenopus. *Proc. Natl. Acad. Sci. USA* 118, e2100342118. 10.1073/pnas.2100342118 33975953 PMC8158011

