# Supplementary material for: Endometrial organoids derived from Mayer–Rokitansky–Küster–Hauser syndrome patients provide insights into disease-causing pathways
Source: Dis Model Mech. 2022 May 10;15(5):dmm049379. doi: 10.1242/dmm.049379 (PMC9118093; doi:10.1242/dmm.049379)
Supplement: Supplementary information [file dmm-15-049379-s1.pdf]

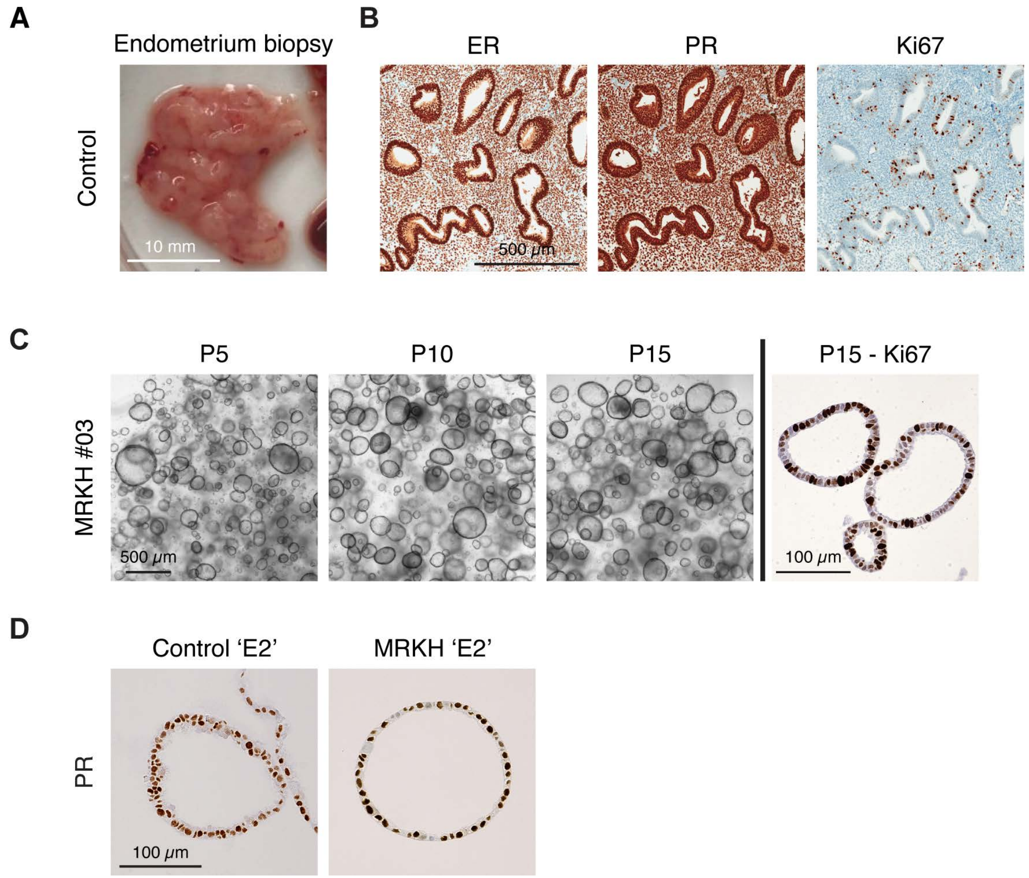

Fig. S1. Long-term 3D organoid cultures can be established from healthy endometrium

A) Endometrium biopsy of a healthy control patient obtained with a Pipelle. Equal parts were used for tissue digestion and subsequent organoid setup as well as pathological characterization; Scale bar = 10 mm

B) Immunohistochemical characterization of control endometrium. Stained was Estrogen receptor alpha (ER), Progesterone receptor (PR), and proliferation marker Ki67. The tissue shows endometrial gland structures with widespread and intense ER and PR expression in glandular and stromal compartments. Proliferative capacity (Ki67) can be seen by in stromal and epithelial cells. Scale bar = 500 μm

C) Bright-field images of endometrial MRKH organoids over different culture passages (passage 5 = P5; passage 10 = P10; passage 15 = P15). Images were taken at the end of each passage before splitting (12-14 days). Scale bar for the brightfield images is = 500 μm. The right panel shows a section of FFP-embedded MRKH organoids from passage 15 which were stained for the proliferation marker Ki67. Scale bar = 100 μm D) Immunohistochemical characterization of Progesterone Receptor (PR) in FFP-embedded control (left) and MRKH (right) organoids under hormonal stimulation with beta-estradiol 'E2'. Scale bar = 100 μm

Disease Models & Mechanisms • Supplementary information

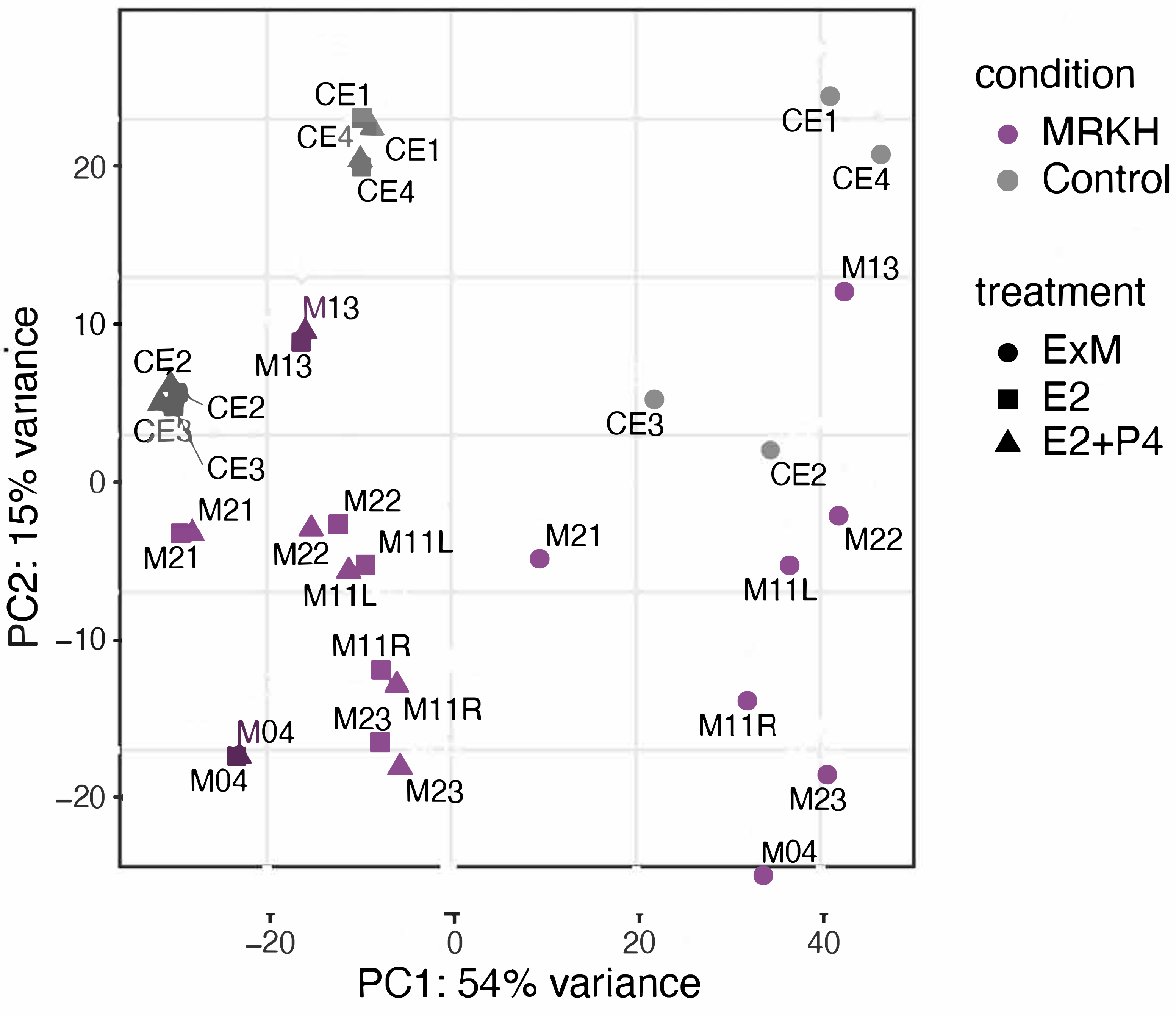

Fig. S2. Organoid expression profiles reflect partitioning of samples according to condition and treatment. Principal component analysis of gene expression profiles for all samples based on the top 500 most variable genes. Axis percentages indicate variance contribution of the first two principal components. Control: organoids derived from unaffected women, MRKH: organoids derived from MRKH patients; ExM: organoids grown in expansion medium, E2: organoids treated with beta-estradiol, E2+P4: organoids treated with beta-estradiol and progesterone.

Disease Models & Mechanisms • Supplementary information

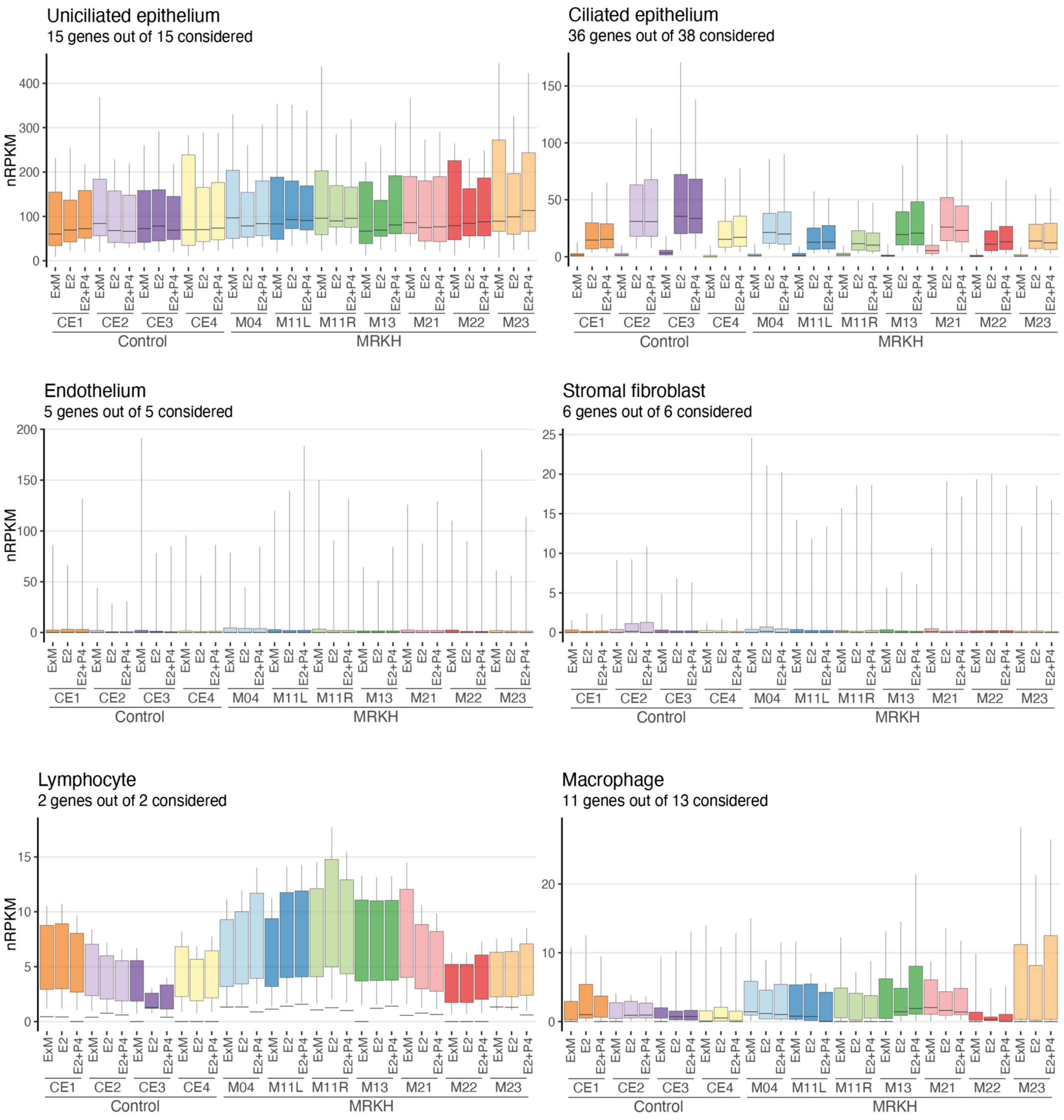
Uniciliated epithelium
15 genes out of 15 considered
Ciliated epithelium
36 genes out of 38 considered
Endothelium
5 genes out of 5 considered
Stromal fibroblast
6 genes out of 6 considered
Lymphocyte
2 genes out of 2 considered
Macrophage
11 genes out of 13 considered
nRPKM
ExM
E2
E2+P4
CE1
CE2
CE3
CE4
M04
M11L
M11R
M13
M21
M22
M23
Control
MRKH

Fig. S3. Epithelial cells dominate cell type-specific expression signature in organoids.

Cell type-specific gene expression per sample for unciliated and ciliated epithelium, endothelium, stromal fibroblasts, lymphocytes, and macrophages. Boxplots show geometric mean as well as 10th, 25th, 75th, and 90th quantile of expression values for all genes grouped based on single-cell reference data of human endometrium (Wang et al., 2020).

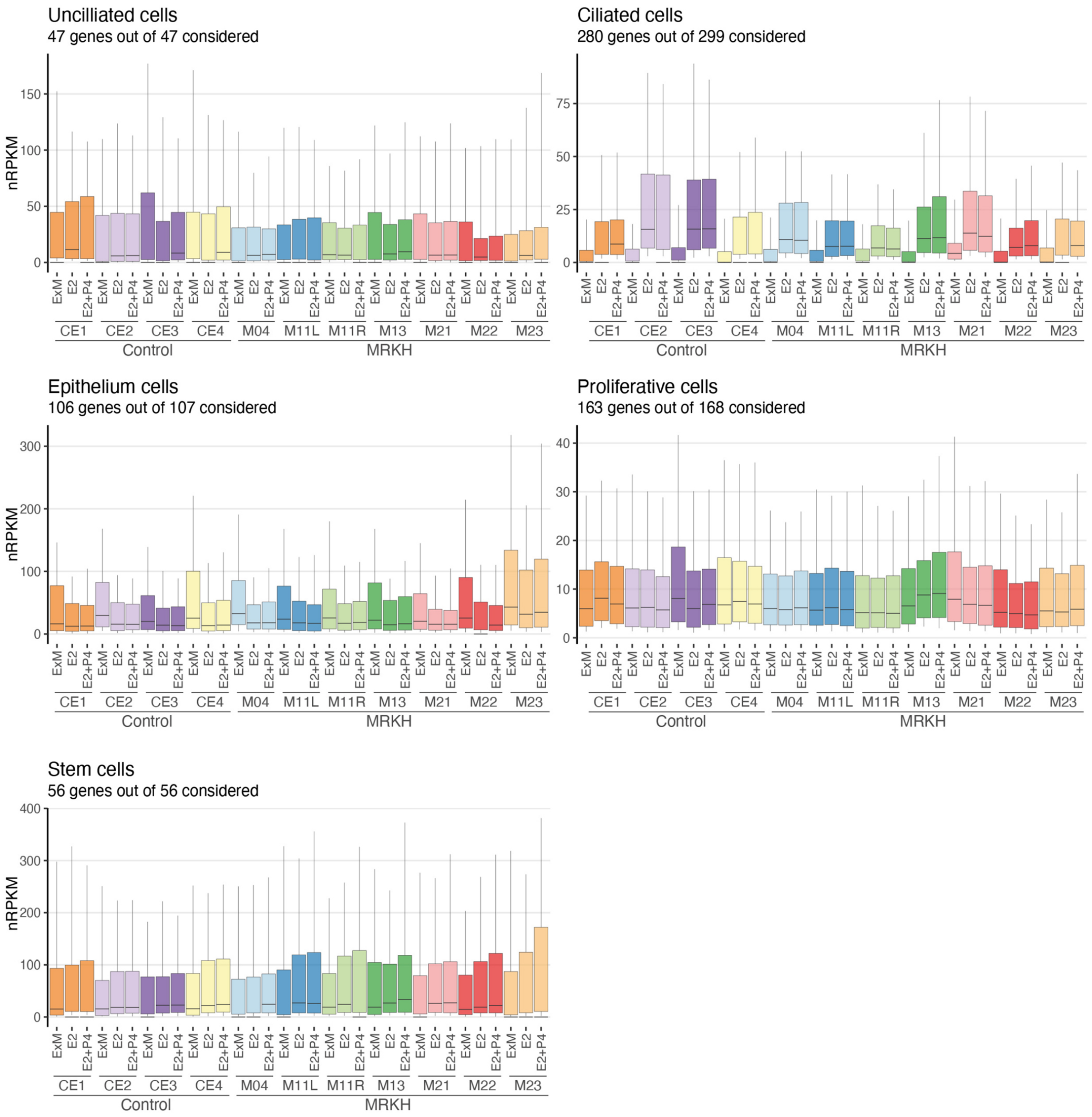

Fig. S4. Ciliated cell-specific genes increase expression upon treatment with steroid hormones.

Cell type-specific gene expression per sample for unciliated, ciliated, epithelium, proliferative, and stem cells. Boxplots show geometric mean as well as 10th, 25th, 75th, and 90th quantile of expression values for all genes grouped based on single-cell reference data of endometrial epithelial organoids of control samples (Fitzgerald et al., 2019).

Disease Models & Mechanisms • Supplementary information

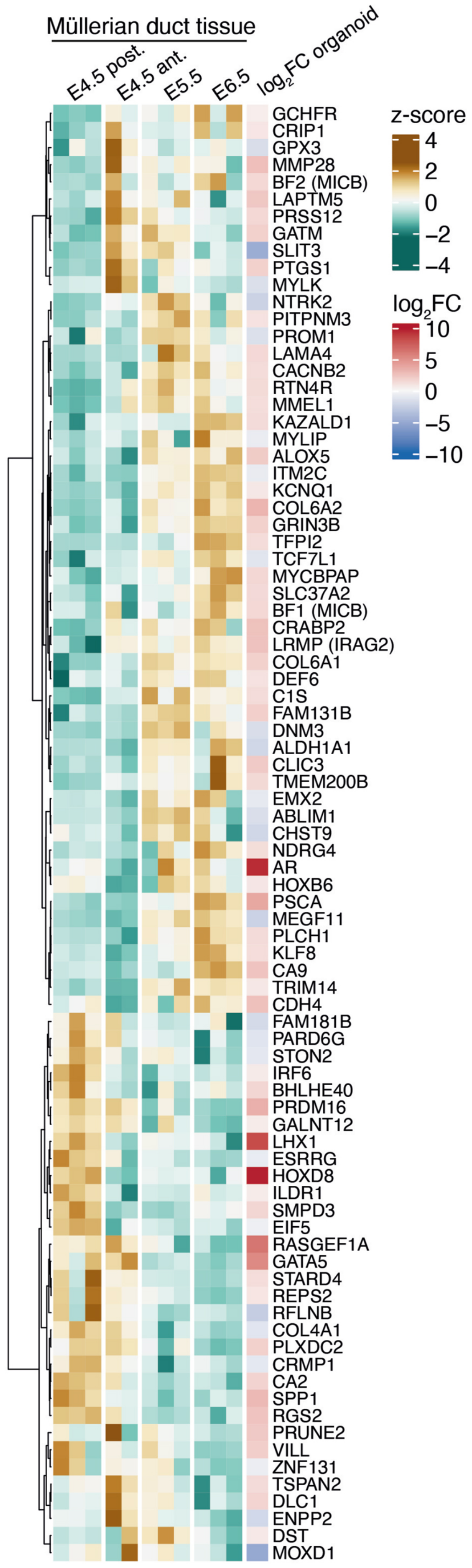

Disease Models & Mechanisms • Supplementary information

Fig. S5. Several differentially expressed genes play a role in the embryonic chicken Müllerian duct.

Z-score heatmap of 85 differentially expressed genes also altered in Müllerian duct development in chicken. Of 492 DEGs, 251 orthologues genes were found in chicken and of those 85 were differentially expressed and had a CPM >5 in one of the static or dynamic transcriptomic changes during duct formation pointing at developmentally regulated genes (Roly et al., 2020). Left part of the heatmap reflects transcriptomic changes in chicken based on data from Roly *et al.* and right part shows expression changes of these genes observed in MRKH versus control organoids.

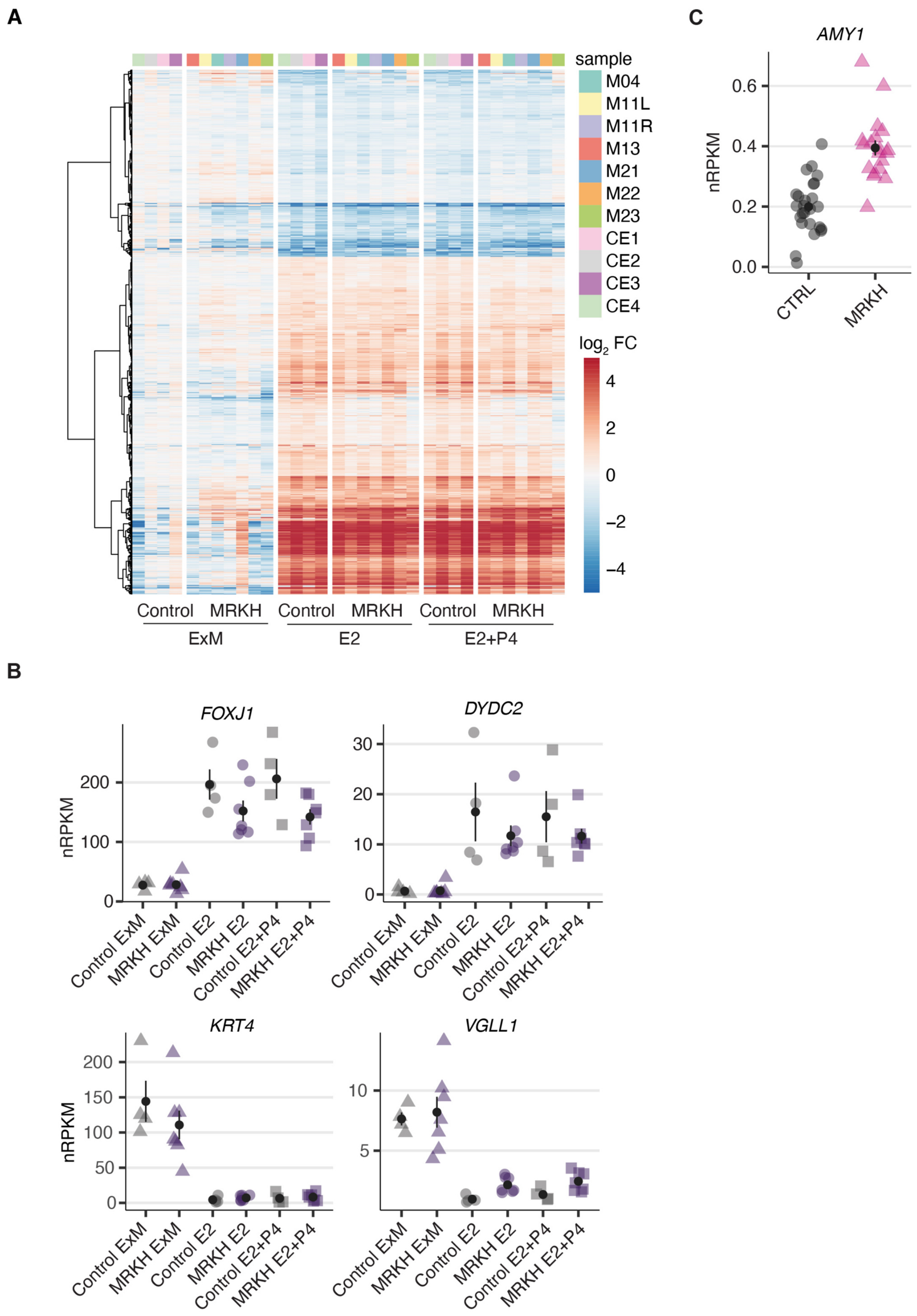

Disease Models & Mechanisms • Supplementary information

Fig. S6. Similar transcriptomic response to beta-estradiol in MRKH and control organoids.

A) Expression profiles (log_2 expression change relative to control ExM group) of 2871 DEGs (union of DEGs comparing E2/ExM in MRKH and control organoids) across all samples. Rows hierarchically clustered by Euclidian distance and *ward.D2* method. Patient origin color-coded.

B) Expression levels of four genes showing pronounced up- (*FOXJ1*, *DYDC2)* respectively down-regulation (*KRT4*, *VGLL1)* upon treatment with beta-estradiol. Plotted as individual data points with mean ± SEM.

C) Expression levels of *AMY1* in primary endometrial tissue plotted as individual data points with mean ± SEM (Hentrich et al., 2020).

Disease Models & Mechanisms • Supplementary information

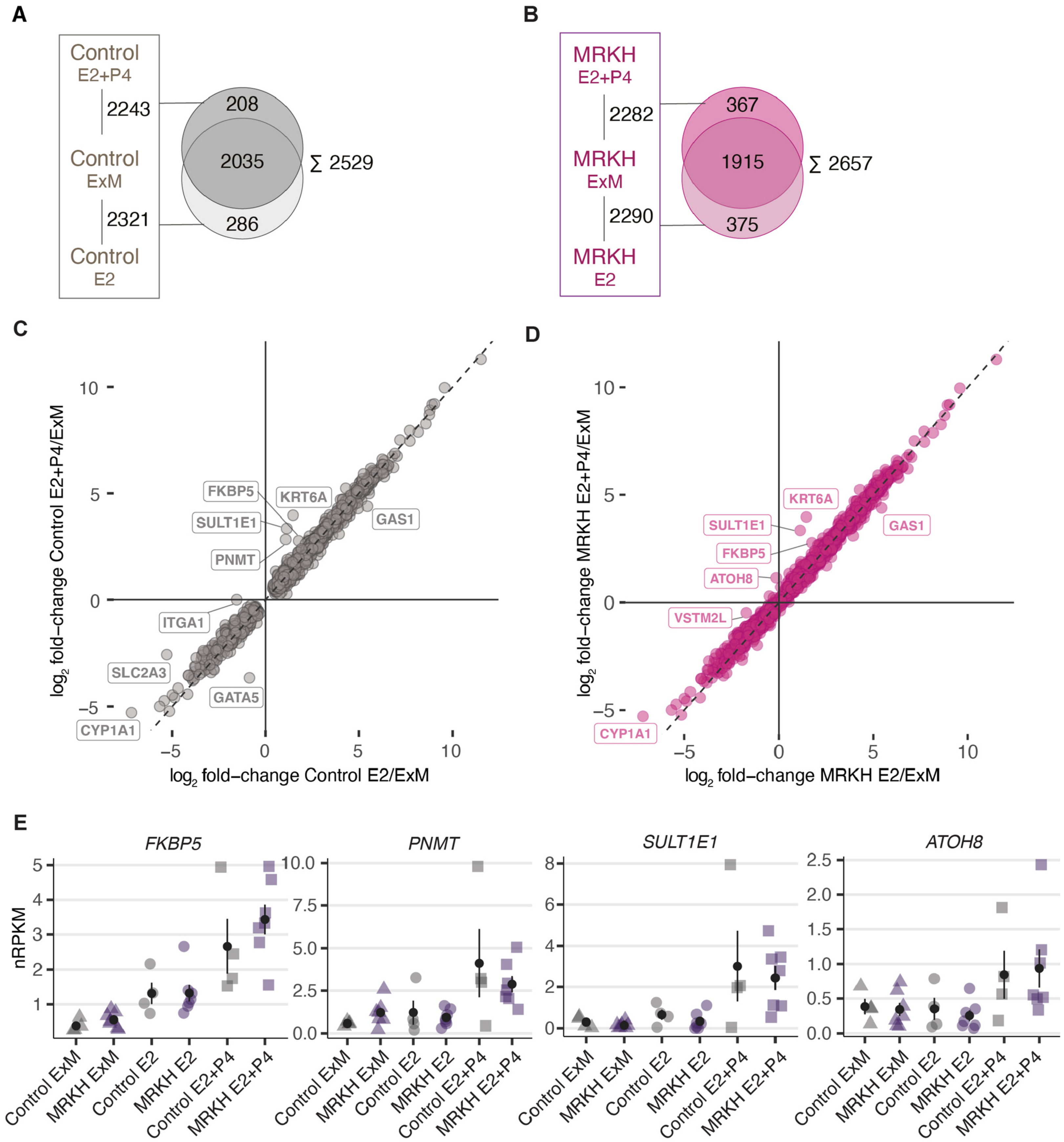

Disease Models & Mechanisms • Supplementary information

Fig. S7. Marginal differences of transcriptomic response to combined beta-estradiol/progesterone treatment versus beta-estradiol alone.

A) Number of differentially expressed genes between control groups upon treatment with beta-estradiol (E2) or beta-estradiol in combination with progesterone (E2+P4). Venn diagram comparing common and distinct DEGs of pairwise comparisons in the right panel.

B) Number of differentially expressed genes between MRKH groups upon treatment with beta-estradiol (E2) or beta-estradiol in combination with progesterone (E2+P4). Venn diagram comparing common and distinct DEGs of pairwise comparisons in right panel.

C) Scatter plot of 2529 DEGs (union of DEGs in A) depicting expression changes of E2/ExM (*x*-axis) and (E2+P4/ExM) (y-axis) in control organoids. DEGs differing between both treatments by more than $|\log_2 FC| > 1$ are labelled.

D) Scatter plot of 2657 DEGs (union of DEGs in B) depicting expression changes of E2/ExM (*x*-axis) and (E2+P4/ExM) (y-axis) in MRKH organoids. DEGs differing between both treatments by more than $|\log_2 FC| > 1$ are labelled.

E) Expression levels of selected genes showing pronounced differential expression upon treatment with beta-estradiol (E2) and beta-estradiol in combination with progesterone (E2+P4), respectably. Plotted as individual data points with mean ± SEM.

Table S1. Demographic and clinical characteristics of MRKH patients

Click here to download Table S1

Table S2. Culture medium for expansion of control and MRKH organoids

Click here to download Table S2

Table S3. List of antibodies used for immunostaining

Click here to download Table S3

Table S4. List of primers for qPCR

Click here to download Table S4

Table S5. GO-term association of the top hundred DEGs

Click here to download Table S5

Table S6. DEGs for all primary contrasts

Click here to download Table S6
